# Masculinity, femininity, and leadership: Taking a closer look at the alpha female

**DOI:** 10.1371/journal.pone.0215181

**Published:** 2019-04-12

**Authors:** Monika K. Sumra

**Affiliations:** Department of Anthropology, University of Toronto Scarborough, Toronto, ON, Canada; Middlesex University, UNITED KINGDOM

## Abstract

An extensive review and textual analysis of the academic and popular literature of the human alpha female was conducted to examine the social construction and expression of the alpha female identity in a small non-random sample of North American women (N = 398). This review revealed 2 predominant alpha female representations in the literature–one more masculine versus one more feminine–and 21 alpha female variables. In this sample of women, the “alpha female” was found to be a recognized socially constructed female identity. Univariate analysis revealed positive and highly significant differences in self-reported mean scores between alpha (N = 94) and non-alpha (N = 304) females for 10 variables including, masculine traits, leadership, strength, low introversion, self-esteem, life satisfaction, sexual experience, initiates sex, enjoys sex and playing a dominant role in sexual encounters, with alpha females scoring higher than non-alphas. The measure of masculine traits was identified as the only predictor of alpha female status as per the multiple regression model. Interestingly, both alpha and non-alpha women scored the same for the measure of feminine traits. Further, both groups scored higher for feminine traits than masculine traits. The results also revealed that neither social dominance nor sexual dominance were predictors of alpha female status which challenge academic and popularized representations of this identity. The results suggest that although the alpha female is often regarded as an exceptional and, at times, an exoticized form of femininity, like other femininities, her identity is marked by contradictions and tensions

## Introduction

Individuals considered leaders in society who occupy the highest positions such as heads of corporations, senior management and those that hold political office are often referred to as “alpha” [1–16]. “Alphas” exercise influence over others, play a lead role in goal-setting, goal achievement, the development of a group or organization, and are regarded as leaders by other members of a group [17]. The term “alpha”, or more specifically “alpha male”, originates from the field of animal behavior and is used as a descriptor for the highest-ranking individual of a social group [18–31]. Popularized narratives and discourse within this context rely on analogies between human and primate behavior [1–4], [16], [32–36].

In the West, alpha women have been described both within the context of masculinity and femininity. Masculine traits such as aggression, assertiveness, academic and professional achievement, confidence, being a supervisor or manager, and exhibiting a “type A” personality [7–11], [37–45] are often used to describe the alpha female. Alpha females have also been described as being “uninhibitedly feminine and sexy” [46], “having a heart” [47], and being a “social lubricator” [48].

Previous research on the alpha female has categorized women as alpha or non-alpha based on assumed aspects of the identity, such as leadership. This approach however, does not allow insight into our understanding of whether women who are classified as alpha, acknowledge or even occupy the alpha female identity. The present research seeks to addresses these gaps in the literature through an examination of the social construction of the alpha female identity in a small non-random sample (N = 398) of women in North America. This examination includes textual and statistical analyses of the qualitative and quantitative data collected from the academic literature, popular media, and most importantly, perspectives of women themselves through focus groups and interviews. The purpose is to gain a more holistic understanding of alpha female and what if anything, distinguishes her from other women.

### Origins of the “alpha” individual

The term “alpha” connotes top ranking status in some kind of social hierarchy [20] and has been studied extensively in the social behavior of animals. This concept has been observed and documented since the 1800’s in a variety of social animals including chickens, wolves, walruses, fish, gorillas, monkeys and meerkats [49–52]. Though alpha status and the importance of the dominance hierarchy or “pecking order” has been discussed by many scholars, it was Pierre Huber, an entomologist, who first related the concept of dominance relationships to social behavior in his work on bumblebees in 1802 [53]. Subsequent research revealed that these relationships were not only orderly, they were also predictable [54]. In the early 1900’s ethologists and comparative psychologists further developed the study of dominance as an integral part of social behavior in which they began to use the terms dominance/dominant and “alpha” interchangeably.

It was Schjelderup-Ebbe’s [55] pioneering work on the social behavior and dominance orders in flocks of chickens, that drew the interest and attention of scholars to the concept of social dominance [53], [56]. Schjelderup-Ebbe [55] found that chickens interacted with one another in either a dominant or subordinate manner. He termed this form of social organization as the “peck order”. His observations revealed that only dominants pecked on subordinates, males dominated females, and older chickens dominated younger ones. What is most interesting is that Schjelderup-Ebbe [55] first described this concept in his work on hens, adult female chickens, in his PhD dissertation in 1921. He used Greek letters to denote hierarchy. The hen with the highest status or peck order, was given the first letter of the Greek alphabet, alpha (α). This appears to be one of the first academic sources in the animal literature that uses the term “alpha” to reference an individual of the highest social status, specifically, describing the alpha female [56]. Despite this, the focus among social behaviorists has been on the alpha male, perhaps because of the underlying assumption that alpha males tend to dominate not only other males, but all females, including the alpha female.

According to Schjelderup-Ebbe [55] the pecking order of chickens and other birds, represented a social system in which some individuals had preferential access to food while others waited their turn. This idea of pecking order, within which flock members gained access to food was soon generalized to other contexts to reflect power hierarchies existing in other social species including nonhuman primates. The term “pecking order” was extended into the concept of male-dominance where the individual in the top spot has priority access to food, mates and other resources [57]. The terms “alpha male” and “pecking order” eventually became common terminology used to describe dominant individuals in social groups [57].

The most well-known example of an alpha-driven group in the animal literature is the wolf pack. In 1947, animal behaviorist Rudolph Schenkel [58], brought together captive wolves from different zoos to create and study “pack” behavior. Schenkel [58] observed that wolves fought each other to gain dominance and establish order in the group. The male and female wolves that came out on top eventually became what he called the “alpha pair,” and claimed exclusive rights to sexual reproduction within the pack.

Social dominance as a form of achieving alpha status, has also been studied extensively by primatologists. Research has examined alpha male behavior in baboons [59, 60], monkeys [61], and chimpanzees [1], [62]. Across the primate literature the alpha male is described as a dominant and aggressive individual with priority access to resources and females for reproduction, is considered attractive and desirable by females, and is more sexually active and reproductively successful than his subordinates [1], [21–30]. Maslow [63] considered male dominance in primate social groups to be analogous to the “peck order” in chickens, believing it to be at the center of all primate relationships [63,64] (Maslow, 1936; Stevenson, 1991). Early research on macaques and baboons also emphasized dominance rank as integral to social cohesion [65]. In 1932, Solly Zuckerman [66] extended the concept of the alpha male and social dominance in his research on captive hamadryas baboons in the London Zoo. He observed that through sexual competition, the strongest male gained primary access to females, food and other resources. Zuckerman [66] asserted that sex was the social “glue” and that male competition expressed through dominance, was the principle that defined their social group [67]. Carpenter [50] also found a positive correlation between rank and sexual activity among communal howler monkeys. However, for this species, he noted that social cohesion was maintained not through male competition but through “cooperation, affiliation, and mutual interest” [67]. Thus, how the alpha male maintains his social status, differs from species to species.

According to de Waal [18], the term alpha female as it is applied to women, originated from the field of animal behavior, specifically nonhuman primate literature. In the nonhuman primate literature, the alpha female has been described as behaving both similarly and differently [18], [38] than her male counterpart. For example, alpha female apes have been described as rarely showing open rivalry for the top spot. Unlike alpha males who exhibit coercive behaviors [38], the nonhuman primate alpha female is described as choosing a more cooperative and communal approach to reaching and maintaining her alpha status [3]. According to Maslow (1940), the techniques and hypotheses that have come from the study of primates, specifically social dominance and dominance rank, including the alpha male or female, can be applied to similar scientific study in humans.

A literature review revealed that reference to the terms alpha male and female used to describe top-ranking individuals in human society in popular literature began as early as the 1930’s. In 1932, Aldous Huxley [68] wrote his famous work, a popular science fiction novel, *Brave New World*. He vividly described a society where people are “decanted” or born in a laboratory into pre-defined social positions in Western society. Each person occupied a prescribed social position or rank exhibiting behaviors associated only with that rank. Like Schjelderup-Ebbe [55], Huxley [68] used the Greek alphabet for the purpose of denoting social rank or position in a social group. Alphas were ranked the highest followed by Betas, Gammas, Epsilons, and Morons. “Alpha” men and women were described as leaders, successful, beautiful, sexually charged, and promiscuous. According to Huxley [68], the existence of a social hierarchy is a necessity for human society; one that is necessary for “happiness and stability” (p. 152). Similar to what has been observed with nonhuman alpha primates, Huxley [68] described alphas as having greater access to resources including, money, sex, and recreational drugs. Huxley’s [68] use of the term alpha as it pertains to humans is significant. Although *Brave New World* [68] is a work of fiction, linking human behavior and social hierarchy to that of primates runs the risk equating alpha humans and alpha primates. Huxley’s [68] use of the term “alpha” and associated traits to describe the “top dog” in human society is a very early example of how primate social behavior was mapped on to human social behavior.

### The alpha woman–dominance, leadership, masculinity, and femininity

Perhaps the concept of the alpha woman had her humble beginnings as a “dominant woman”. In his 1939 publication *Dominance*, *Personality*, *and Social Behavior in Women*, Maslow [69] was specific in his description of the traits of dominant women or what he termed, “dominance-quality”. His research was based on in-depth interviews with 130 women and 15 men aged 20–28 years. The women were middle-class, went to college, 75% were married, 75% were Protestant, 20% Jewish, and 5% were Catholic. Maslow [69] stated that high-dominance women would make great leaders, though not every dominant woman would become one. He also described dominant women (high-dominance feeling) as rarely embarrassed, self-conscious, shy, or fearful compared to women who were not dominant (low-dominance feeling). According to Maslow [69] dominant women have more self-confidence, higher poise, prefer to be treated like a “person” and not like a “woman”, prefer independence and “standing on their own feet”, lack feelings of inferiority, and generally do not care for concessions that imply they are inferior, weak or that they need special attention and cannot take care of themselves. Maslow [69] also stated that being a dominant woman does not preclude her from behaving like a “conventional”, or traditional woman which implies that the traits he describes are inherently “unconventional”. Maslow’s [69] work also revealed that dominant women do not behave in a dominant fashion exclusively or occupy a leadership role in all social domains. Though not explicitly stated, given current notions of the alpha woman one can easily extrapolate Maslow’s [69] “dominant woman” to the “alpha woman”.

The alpha female has often featured prominently in the popular media as a type of female identity [7, 15, 42, 43, 45, 70–72]. During the 1980’s the concept of alpha woman began to gain momentum with feminist and activist Betty Friedan’s 1981 book, *The Second Stage* [73]. In a follow-up edition of this book, Friedan [74] modified the introduction to include some of her experiences in the early 1980’s. She describes an “unusual” meeting she attended a week before the October 29, 1980 US election about the “crisis of leadership in the U.S. which may be less about the particular leaders we have than the style of leadership we have come to expect” [74]. The solution she states is to “balance the dominant Alpha, or masculine leadership style, with the Beta, a more feminine leadership style” [74]. Citing research by Peter Schwartz of the Stanford Research Institute, “alpha-style leadership in our [Western] society is considered more masculine. It is based on rational, analytical, quantitative thinking, is more aggressive and direct [74]. These references present the concept of an “alpha leader” as an inherently male concept and only occupied by men. Further, these references serve to reinforce traditional Western roles of masculinity and femininity. Also, in her book, Friedan [74] stated that Schwartz’s research also revealed that “younger women moving up in the traditionally male-dominated fields of engineering and business now test higher than males in the dominant male Alpha mode” [74]. Schwartz also stated that this “reversal” would be “dangerous to society”. Some have described such women as “original” or “stereotypical” alpha females–“driven, unemotional, and not letting anyone stand in her way” [47]. Though not explicitly stated by Friedan [74], the inclusion of this interface with Schwartz may suggest that the idea of alphaness, as a male concept, is critical cultural terrain for discussions around gender, nature, leadership, and power.

Perhaps one of the most influential figures to contribute to the understanding and popularization of the human alpha male and alpha female is anthropologist and primatologist, Franz de Waal who contends that the term “alpha male” was not actively used outside primatology until after the publication of his book *Chimpanzee Politics*: *Power and Sex Among Apes* in 1982 [1, 18]. *Chimpanzee Politics* [1], a study of male dominance and reproductive strategies in the Arnhem Chimpanzee colony, has been widely referenced by primatologists in the study of nonhuman primate social behavior [75–79], as well as a much broader audience including politicians and business leaders for the insight it offers into the understanding of human social hierarchy and behavior [80–82]. Applying primate models of behavior to humans in this manner runs the risk of both the misuse of primate studies and simplification of human behavior.

For example, in 1995, Newt Gingrich then Republican Speaker of the House of Representatives in the United States, placed *Chimpanzee Politics* [1] (1982) as one of the 25 books on his recommended reading list for incoming young congressional Republicans [83]. The term alpha male also received notoriety during the 2000 US election campaign. There were rumors that then presidential candidate Al Gore’s image consultant, Naomi Wolf, had told him that he is a beta male who must fight [Bill] Clinton’s alpha male for dominance [6]. However, though she acknowledged that she did mention alpha versus beta males, she did so only in conversation [84]. Wolfe clarified her role in the Gore’s campaign in a later version of the New York Times article covering this story. She was a consultant to candidate Gore on women’s issues and outreach to young voters, and not his image consultant [84]. What is interesting is how the terms “alpha" and “beta” were used to describe characterizations of leadership at the highest level of office in the United States. The term “beta male” was used in a sense, to question the leadership capability of candidate Gore. This example suggests that the blind application of primate studies to human behavior has the potential to influence various institutions, including politics. What needs to be considered is that such presumptions can impact decision-making, in this case, who one may, or may not vote for. The result, our not-so-real understanding of human behavior can have real impact on social, political, and even economic factors that influence the lives of many.

### The alpha female in the 1990’s and 2000’s–persuasive and pervasive

During the late 1990’s and early 2000’s the notion of human alpha female began to gain momentum. The search terms “alpha woman” and “alpha female” in Google Ngram Viewer (a web application that displays the usage of words and phrases over time sampled from millions of books scanned by Google), show a spike in use of these terms during this period in popular articles and books and in research in the fields of psychology, the scientific study of sexuality, as well as in leadership studies. Articles and books during the early 2000’s on this topic question her existence [85], confirm her existence [86], and describe her as a “one-off” character, who defies “categorisation”, one who is “deadlier than the [alpha] male”, [87]. In research on alpha women and sexual fantasies, Hawley and Hensley [37] suggested that the ‘alpha female’ may rival alpha men in terms of “behaviors and motivation”.

Research on the human alpha female has focused primarily on leadership [7, 8, 10–13, 88, 89]. In this research women who hold a leadership position in student and business organizations are labelled “alpha female”, a “special kind of leader” [11]. Some authors have argued that the emergence of the alpha female identity has been driven by the changing context of leadership, specifically with women taking on more leadership roles, as well as the shift in women’s social roles over the past decade. This, as some suggest, has led to a rethinking of gender-role stereotypes in the West [7, 10, 11].

In Kindlon’s [7] study, a total of 113 girls across 15 North American schools were recruited to participate in a study of alpha girls. Girls who had the highest rank in social groups such as class presidents, captains of basketball teams, and other social group leaders, were identified as alpha. Other alpha-status inclusion criteria included a GPA of 3.8 or higher, a minimum of 10 hours per week participation in extracurricular activities in or out of school, a high achievement motivation score, and a high self-rating for dependability. According to Kindlon [7], an alpha girl is an assertive, decisive and a confident female cognizant of her life choices; a person ready to take risks and willing to "transcend the barriers of race and class" (p. xvii). Kindlon [7] discusses the emergence of this alpha female identity in the context of the major gains made by women in the West such as the right to vote, to make reproductive choices and the right to participate in athletic sports previously not accessible to them. According to Kindlon [7] what was most noteworthy in his results was that alpha and non-alpha girls were similar in many ways. Kindlon [7] concluded that though an alpha girl is a leader, she is also a female in a “generation on the rise” [7] (Kindlon and that “alpha girls”, in some sense, represent a whole generation of females.

Ludeman and Erlandson have studied the concept of the alpha male leader extensively. According to Ludeman and Erlandson [8, 88, 89] (2007), men in leadership positions are alpha males described as “well-balanced human beings in full command of their strengths, are esteemed by colleagues, revered by employees, and adored by Wall Street” (p. 38). They further state that alpha men “inspire fear and resentment rather than trust and respect” [89]. In their comparative research on male and female leaders, the authors found that male leaders scored significantly higher than female leaders on all the attributes that they define as “alpha” (e.g. charismatic leadership, dominance, confidence, aggressive, competitive, persistent, far-sighted, and bold) [89]. Their analysis revealed that alpha traits are correlated with being a male who fits into one of 4 alpha groups; 1) commander, 2) visionary, 3) strategist, or 4) executor [89]. Though the authors did not conduct direct research on the alpha female they state that women possess the same fundamental traits as alpha males [89].

In 2009, colleagues Rose Marie Ward (health psychologist), Donald DiPaolo (Professor and leadership researcher), and Halle Popson (health promotion), were the first to conduct research on the alpha female identity. Their first study was an examination of the alpha female identity as a measure of leadership among 13 undergraduate women at a midwestern university in the United States. Only women who were well-known on campus and held a leadership position in a student organization were recruited. Data on their leadership characteristics, situations where these were displayed, whether they viewed themselves as a leader, and alignment with a specific definition of the alpha female–“a woman who reports being a leader, feeling a sense of superiority or dominance over other females, having others seek her guidance, feeling extroverted in social situations, believing that males and females are equal, feels driven, and is highly self-confident” [10] were collected. The authors developed this definition from the leadership and dominance concepts presented in the alpha male literature and Kindlon’s [7] work. According to Ward et al., [11] this definition encompasses the aspects of leadership that are endorsed by alpha females [11]. The results of their study revealed that alpha females come from a nurturing family environment and had role models who taught them that being female was either a non-issue or an advantage [10]. Further, Ward et al. [10] also stated that it is this teaching that has facilitated the ability of alpha females to “push boundaries”.

In their second study, *Defining the Alpha Female*: *A Female Leadership Measure*, Ward et al. [11] develop and present a 14-item measure of an alpha female personality. The development of the Alpha Female Inventory or AFI [11] was guided by the alpha female definition developed in their previous work. According to the authors, the AFI can be used to identify alpha females [11]. The AFI comprises three subscales: the AFI-L (leadership), AFI-S (strength), and the AFI-LI (low introversion) [11]. The AFI-L assesses a woman’s desire to be a leader, to be dominant and assertive, while the AFI-S measures a woman’s superiority and perceived strength, and the AFI-LI measures extroversion. The authors argue that low levels of introversion are synonymous with being more extroverted, social or outgoing [11]. Women who score high on each of the subscales, that is, they “agree” or “strongly agree” with the items, are categorized as alpha female, and all others as non-alpha females.

The results of Ward et al.’s [11] study revealed that alpha females were not different from non-alphas in terms of year in school, mother’s and father’s education level, family income or age, though they did have significantly higher GPAs. Though alpha females had higher levels of leadership characteristics and more masculine gender-role characteristics than non-alpha females, there were no differences in self-esteem and emotional intelligence [11]. The authors did suggest however, that additional research that examines leadership with respect to general dominance measures and determining whether there are more alpha females in college in comparison to the community at large is needed [11].

More recently, Poduška [12] translated and performed a validation of the Ward et al.’s [11] AFI in their study of Croatian female university students. Although the composition of the leadership components of the AFI were different than Ward et al. [11], Poduška [12] found that alpha females evaluated themselves significantly higher on measures of self-efficacy and self-monitoring, and they engaged in more leader-like behaviors in the student environment.

Departing from previous research that focused on alpha females as student leaders in a public school or university setting, Moncrief [13] used the AFI to gain insight into how being a “veteran” alpha female leader is influenced by minority identity (ethnicity), leadership experience (modality, display and presumption), and duration (10 years or more in a leadership role). Recruitment for their study was restricted to women who self-identified as leaders and had a minimum of 10 years of experience in a leadership role [13]. The 12 women who participated in their study had leadership experience ranging from 10 years to 40 years, held a variety of leadership positions, came from different industries and had different ethnic backgrounds [13]. All 12 women were self-identified leaders and scored as alpha on Ward et al.’s [11] Alpha Female Inventory (AFI).

Ward et al. [11] have put forth the AFI as an appropriate measure to identify alpha. It is important to note however, that the underlying assumption of the AFI is that women who are in leadership roles in different contexts are alpha women and as such, the AFI [11] does not measure the alpha female construct necessarily; it measures female leadership. Ward et al. [11] examine the relationship between self-identified female leaders with leadership characteristics consistent with the male leadership literature. These include qualities such as emotional intelligence, masculine and feminine gender-role traits, and self-esteem [11]. As a result, other forms of the alpha female leader, such as a female model of leadership, may not be easily discernable. Additionally, other characteristics or traits related to the alpha female identity such as dominance (sexual and social), collaboration and affiliation and life-satisfaction that may also contribute to the alpha female construct are not explicitly incorporated in the AFI [11]. Inclusion of such traits may also contribute to measures such as the AFI [11] in identification of alpha females. Ward et al.’s [11] AFI inventory is invaluable in identifying alpha females in terms of leadership, strength, and low introversion. What may add greater insight is an investigation that examines whether women who identify themselves as alpha female also express the components of the AFI [11]. An examination of the relationship between self-identification as an alpha female and traits related to the expression of the alpha female identity, including those presented by Ward et al. [11] would provide additional insight into our understanding of the alpha female identity. In the present study I examine the alpha female as a potential form of female identity and ask women themselves whether they identify as alpha or not. Thus, though self-identification is a different approach from previous research that has used the AFI [11] to identify alpha females in different populations and contexts [11–13], this approach offers the opportunity to evaluate the alpha female as potential form of female identity which may also include the expression of traits presented by Ward et al. [11], adding to the research in this area.

Like his earlier metaphorical comparisons of the alpha male primate to alpha men, de Waal [3] also engaged in similar comparisons of alpha female primates to alpha women during the 2000’s. In his 2007 popular article, *Alpha Females I Have Known*, de Waal states that ‘alpha female’ refers to women who “are in charge”–through “flirting” and “dating on their own terms” [3]. He also states however, that characterizations of alpha women as “loud-mouthed and controlling, with no patience with deviating opinions”, do not present an accurate picture [3]. According to de Waal [3] (2007), alpha female means the same as alpha male–“the highest-ranking member of one’s sex with all the traits and advantages associated with it” [3]. Though he states that despite the underlying assumption that the highest rank is about “being the strongest and nastiest”, he also states that being an alpha female is about connection and relationships [3]. De Waal [3] likens alpha female chimpanzee traits such as problem-solving, being “sweet, calm, and reassuring” which he suggests may be why we do not “notice” her status [3]. According to de Waal [3], several alpha female primate attributes can be applied to alpha women. He cites age as contributing factor to alpha female status–older women in post-reproductive state such as Indira Gandhi, Angela Merkel, and Margaret Thatcher. He also states that female solidarity is the “key” to alpha female leadership and that an alpha female needs to rise above others [3]. He states however, that unlike alpha males, alpha females should not have sex appeal–not be “overly attractive”. His has numerous publications which compare other aspects of primate social behavior to human social behavior such as empathy [90], morality [91], conflict resolution [92], and altruism [93].

### Alpha female sexuality

Though de Waal [3] contends that an alpha female should not be “overly attractive” and should not have “sex appeal” like the alpha male [3], more recently, depictions of the alpha female have expanded to include physical appearance as well as sexuality as part of how she expresses her identity. Across the popular media, when compared to other women, the alpha female is often described as taking more pride in her appearance, considered more attractive and desirable by men compared to other women, superior in sports, having a large social network, and is not afraid to be “uninhibitedly feminine and sexy” [7, 15, 42, 43, 70–72]. Topics in the popular media vary from how to become an alpha female [9, 15, 41, 43, 70–72], to how being an alpha female makes you “sick” [46, 94–96]. The alpha female has also been described as a femme fatale or vixen, a successful leader, a harlot, a high-heeled powerhouse, intelligent, sophisticated, cut-throat, aggressive, confident and collaborative [7, 15, 42, 43, 70–72]. While some popular narratives depict her as being “unable to love” [45], others say the alpha female may have a “heart” [47]. She has also been described as a powerful woman who is an “adulteress” and sexual predator [97] who is “truly sexy” and “passionate about sex” [46, 98]. What is interesting about her sexuality is that much of the discourse on the alpha female to date portrays her as heterosexual. Though some suggest that the alpha female’s sexuality is not limited to being “strait” [46] there appears to be minimal engagement in this particular discourse both in popular media and academia. The term alpha female also comes up in the bullying literature. Here, she is referred to as a “bully” who manipulates others through fear and threats [99]. Psychologist Dr. Littlemore states that “there tends to be one main type of girl bully–a strong-willed alpha female” who gathers her pack. According to Littlemore [99], this type of alpha female is desperate to establish and maintain a role at the top of the social hierarchy.

Such popularized and polarized notions of the alpha female have been rejected by some women as being an inherently “male” concept and embraced by others who regard the alpha female as a meaningful form of female identity that expresses both the masculine and the feminine. Today’s alpha women according to some, are growing “softer around the edges” both “metaphorically and literally” [47].

### Social Dominance Theory (SDT)

Social Dominance Theory (SDT), a theory of intergroup relations focused on the maintenance and stability of group-based social hierarchies [100]. Social Dominance Orientation (SDO) is a belief system that represents a preference for a hierarchical society in which some groups are more deserving of higher status than others [101]. The Social Dominance Orientation Scale (SDOS), a concept introduced by [40], reflects one’s approval of hierarchical and dominance relationships between social groups regardless of whether or not one’s ingroup is in a dominant position [102]. The SDOS has been used as a validated measure of social dominance in humans [40, 100–103]. Though the SDOS has been predominantly used in research to evaluate discrimination, inequality, and political affiliations [40, 100–104], it serves as a viable instrument to measure the degree to which women in the present study group, may or may not feel that alpha females as a group, are superior to non-alpha females–whether the alpha female identity is indeed, a value laden-identity. As such, social dominance orientation may offer partial insight as to what may or may not be at stake for women who do not identify as alpha. The SDOS is used as an index of social dominance in the present research.

### The alpha female–a social construction

Female identity is a form of social identity that refers to the meaning women attach to their membership in the category “female” [105, 106]. Prevailing narratives and the discourses surrounding the alpha female as an archetype of female identity present her as enigmatic. Is she a “masculine” female or a “feminine” female? To gain insight into this question however, it is necessary to understand how ideas about gender become part of our everyday lived experiences, and this begins with some background on how female identity is socially constructed.

Social constructionism theory proposes that everything people come to know or see as reality is partly, if not entirely, socially situated. A social construct is ontologically subjective in that the construction and continued existence of social constructs depend upon the collective agreement, imposition, and acceptance of such constructions [107]. Perhaps the best example is the concept of race. “Race” is not biological but rather is “real” only as a social construct. It does not exist in any ontologically objective way; however, it still “exists” and is “real” in society. “Race” is a social construction with real consequences and real effects [108]. Social constructs shape the way we see ourselves and others [107]. Like race, the alpha female as a social construct can be regarded as “real” if there is collective agreement and acceptance of the identity. The idea that the notion of the alpha female as a socially constructed identity therefore, does not diminish its sense of reality. A social constructivist approach therefore, lends itself to examination of the alpha female identity. When it comes to the alpha female and gender however, it gets a little more complicated.

### The alpha female and gender

Earlier theorists have presented categories of “women/women” as singular and homogenous however, as women have entered the workforce these categories have become varied resulting in the emergence of different archetypes of female identity [109], such as the “alpha female” or “alpha woman”. Both these terms are used interchangeably in popular and academic courses and predominantly refer to women who are born biologically female and exclusively heterosexual. Academic and popular discourses surrounding the alpha female identity largely reference characteristics or traits that are based on traditional gender roles of males and females in Western society. However, previous research has predominantly focused on gender differences between men and women and alpha male/masculine traits. As such, ideas, and by extension, research that focuses on a human alpha identity tends to be about alpha males rather than alpha females. For example, previous work has shown that collaboration in the workplace has a gendered component. Senior males are said to create highly competitive working conditions, argue about individuals that are junior to them, and have difficulties accepting challenges from them [110]. When it comes to risk-taking behavior, a behavior regarded as “an attribute of the masculine psychology” [111] previous work has shown that women are more risk-averse than men. For example, males are more likely to take risks than females [112]. Risk-taking behavior is considered an outcome of competition—competitions forces dominant individuals to engage in risk-taking in order to attain their positions of power [112]. Similarly, women have been shown to shy away from competition while men embrace [113] and also exit situations of conflict when the cost of this exit is small [114].

Research has also shown that women are more altruistic than men [115,116]. This is important as irrespective of whether women identify more with masculine or feminine traits, by virtue of being female, the expectation in Western society is that she will still engage in altruistic behaviors [115]. As women disproportionately occupy social roles that require cooperative, communal, and sacrificing behavior, failure to engage in such behaviors can result in negative consequences for them [115]. Less altruism is considered a “male” attribute because it is disfavored by both masculine gender roles which involve power, dominance, and independent self-interest, and the general tendency to make people consider strategic self-interest [115].

Women have also been found to be more harm-averse [117] and more honest [118] than men. For example, research has demonstrated that women tend to embrace deontological ethics more than men when faced with personal dilemmas [117]–that is women are more likely to be concerned with what people do rather than with the consequences of their actions. Similarly, research has also shown that when it comes to lying, men are more likely to tell “black” lies which, come with benefits for the liar and someone else, while women are more likely to tell altruistic “white” lies, which benefit another person at their expense [117].The reluctance and/or lack of women in traditionally “masculine” jobs may also influence our assumptions that human alphas are more likely to be male than female. For example, research that examines women’s experiences in “masculine” jobs, such as in the military has shown that negative consequences can occur for such women for example, less perceived family supportive supervisor behavior, and as such, women are less likely to pursue masculine occupations because they perceive that these job are not open or “available” to them, or that there is an expectation that such jobs are “men only” jobs [119]. Research has also shown that women’s disadvantage is greater at higher organizational levels in corporate law firms, limiting the possibility of internal promotions, though this was not shown to be the case for women who are hired outside an organization [120].

These examples suggest that suggest that gender differences in the expression of alpha related traits may partially explain why research has predominantly focused on alpha males rather than alpha females. However, given that in recent decades women’s social roles in the West have changed significantly and that today, more women are employed, educated and have taken on senior leadership roles in their vocations, quashing earlier stereotypes of women being passive, non-competitive and non-progressive [11], a deeper understanding of the alpha female’s gender has become warranted.

Academic and popular discourses surrounding the alpha female identity largely reference characteristics or traits that are based on traditional gender roles of males and females in Western society. Gender identity reflects a person’s understanding of oneself in terms of cultural definitions of female and male [121]. In Western societies, the gender binary represents a system in which a society splits its members into one of two sets of gender roles, gender identities, and associated attributes based on the genitalia an individual is born with–“two discrete sexes and two distinguishable genders because our society is built on two classes of people, women and men” [122].

Although it is recognized that gender and identity do not necessarily fit neatly into one particular “box”, that is gender is not necessarily easily discernable based on biological traits, there is still value in examining the alpha female identity through a gender binary lens. More specifically, such an examination will allow for a deeper understanding of variability in the meanings and practices of being female [123]. Examining this variability within the category woman, may yield to the emergence of a different gender, one that is perhaps at once both masculine and feminine or neither. Thus, it can be said that gender, and variations on how to be a woman, specifically, are socially constructed and attached to sexed bodies and is nonetheless interesting and necessary to understanding the variation within gender categories. As Lorber [122] argues, though differences exist between groups, more often than not, more significant differences exist within groups themselves [122]. A social constructivist approach to identity allows for an examination of gender identity that extends beyond categories of masculine and feminine, problematizes them, and provides the framework within which to examine the variation within gender categories themselves, as well as where they intersect, overlap, or become blurred. A social construction perspective thus, provides an optimal framework within which to examine the alpha female construct as there is the potential to understand it as a variation of the category “woman”. A social construction perspective also suggests that it might be more useful to group patterns of possible masculine and feminine behaviors and examine these among women who are most likely to exhibit them–for the present study, those are women who identify themselves as “alpha female”, rather than starting with a presumed dichotomy [122]. Such a research approach of the alpha female identity has not been made to date but is undertaken in the present research.

In 1974, Sandra Bem developed the Bem Sex Role Inventory (BSRI) [124] challenging the biological innateness of masculine and feminine traits and argued that such conceptualizations are culturally prescriptive–how men and women “should” act [124]. The scale reflects what Americans in the 1970’s considered were masculine traits such as aggression and independence, feminine traits such as being affectionate and sympathetic, and gender-neutral traits such as happy and tactful [124]. According to Bem [124], the Western sex-role dichotomy does not consider two very important things. First, depending upon the context, individuals may be both masculine and feminine in the expression of their gender, and second, that “strongly sex-typed individuals might be limited in the range of behaviors available to them” [124]. For example, a person with a highly masculine self-concept might inhibit behaviors that are considered feminine, and a person with a highly feminine self-concept might inhibit masculine behaviors [124]. According to Bem (1981), sex typing refers to “the process by which society transmutes “male” and “female” into “masculine” and “feminine” [125]. The BSRI [124] allows researchers to assess a third expression of gender, androgyny, a person who is scores high in both masculine and feminine traits [124]. An androgynous individual is comfortable engaging in both masculine and feminine behaviors [124], allowing for a more fluid and flexible expression of their gender unrestricted by traditional Western gender role expectations [126]. Since the 1970s, the original 60-item BSRI [124] and shorter versions have been and continue to be used in many studies as a reliable measure of gender identity across various countries, cultures, ages, and transsexual groups [127, 128]. For example, Gomez-gil et al. [127], used the femininity scale of the BSRI to evaluate differences in the sex-role identification of Spanish transsexuals and non-transsexuals. They also found that male-female and female-male transsexuals score as a function of their gender identity instead of their anatomical sex [127].

Despite criticisms of the scale citing changes in Western perceptions of masculinity and femininity [129], and development of similar scales [130], the BSRI [124]is still extensively used in psychometric studies and other research [131, 132] to measure self-attributed gender-stereotyped personality traits in the West [130, 133, 134]. The BSRI [124] It has also been used in previous research on the alpha female [11]. As such, the BSRI [124] presents itself as an integral tool in the examination of the expression of the alpha female identity. For the purposes of the present research shortened versions of the BSRI-M, BSRI-F, and BSRI-N were developed based on traits that aligned with the current narratives, discourse and research on the alpha female.

### Self-categorization

The concept of “recognition” in social identity theory, specifically the degree to which a group accepts a social identity, and the degree to which non-group members recognize it [135], provides the framework within which to identify the alpha female as a distinct category. According to Tajfel and Turner [136], one aspect of self-categorization theory is that it represents the degree to which a person feels committed or attached to a specific group. The concept of recognition allows for a deeper examination of the extent to which a woman is attached to the category “alpha” as well as the degree to which she feels committed or attached to the associated traits themselves [136]. Thus, asking whether women identify themselves as alpha female provides the opportunity to better understand the alpha female as an accepted form of female identity.

### The Alpha Female-Feminine (AFF) and the Alpha Female-Masculine (AFM) hypotheses

An extensive review and textual analysis of the academic and popular literature, as well as information collected during focus groups revealed two competing conceptualizations of the alpha female in Western society–a more “masculine”, and a more “feminine” alpha female. To examine the two competing conceptualizations, two hypotheses were developed and tested- the Alpha Female Masculine (AFM) and Alpha Female Feminine (AFF). These hypotheses allowed for the examination of the relationships between two often presumed and culturally endorsed gender categories (masculine and feminine) of the alpha female. For the purposes of the present research a shorter version of the masculine, feminine and neutral traits consistent with current discourse, narratives and academic research on the alpha female were developed. These are represented as the BSRI-M (masculine traits), BSRI-F (feminine traits), and the BSRI-N (neutral traits). It is predicted that if the Alpha Female-Feminine Hypothesis (AFF) holds true, feminine traits as indexed in the BSRI-F predict alpha female status, and that compared to non-alpha females, alpha females will report having larger and more diverse social networks and being more collaborative. If the Alpha Female-Masculine Hypothesis (AFM) holds true, masculine traits as indexed in the BSRI-M will predict alpha female status, and that compared to non-alpha females, alpha females would report, higher social dominance orientation, higher life satisfaction, being stronger, being less introverted, being a leader, be more sexually experienced, have sex more frequently, initiate sex, play a dominant role in sexual encounters, and enjoy sex more.

## Methods

### Textual analysis

The present study which was carried out from June to December 2015, began with an initial examination of the results of the search terms “alpha woman” and “alpha female” in Google Ngram Viewer (S1 and S2 Figs), a web application that displays the usage of words and phrases over time sampled from millions of books scanned by Google, and was followed by an in-depth literature review. Data collected from this review were used to conduct textual analyses to gain insight into the social construction of the alpha female identity. The purpose was not only to analyze the discourse and narratives but also to assess what the alpha female identity produces for women in terms of traits, behaviors, beliefs, and practices. Qualitative data collected from popular media such as blogs, men’s and women’s magazines, books, and webpages, as well from academic sources including the leadership and biobehavioral literature (animal, nonhuman primate, and human), and from focus groups and interviews were analyzed using QSR-NVIVO v10.2 [137] software. NVivo is used to gain insights and facilitate the interpretation of unstructured and qualitative data such as interviews, open-ended survey questions, articles, social media and web content [138]. The coding strategy developed for data analysis included using nodes to identify themes within the data collected from participants. The textual analysis was informed by open coding, with emergent tree nodes outlining broad themes and child nodes allowing for more in-depth interrogation of the data. Queries on key words and themes were used to analyze the data.

In addition to using NVivo, other manual methods including content analysis to identify, enumerate and analyze occurrences of specific alpha-related themes, and interactional analyses, to identify and describe the interactions between focus-group participants in order to gain insight into alpha woman related topics, themes, associated words and phrases, formed the framework of the textual analysis [139]. The textual analysis allowed for a broad understanding of the social construction of the alpha female identity which facilitated identifying common, and recurring alpha female themes needed to develop a working description that would guide the research. The analysis revealed 8 themes and 21 potential alpha female predictor variables or traits from which, a working definition of the alpha female was developed. To validate the themes an additional 4 focus groups and 10 interviews were conducted. Open-ended questions to generate general discussions on the subject of the alpha female were asked and notes were taken. Content analysis performed on the data collected from these focus groups and interviews revealed highly similar themes that corresponded with the original 8 themes. To develop a measure of these traits a study of pre-existing survey instruments was conducted. Some variables were assessed using pre-existing validated instruments. Components of the BSRI [124] were used to measure masculine traits (BSRI-M) feminine traits (BSRI-F), and neutral traits (BSRI-N). Ward et al.’s [11] Alpha Female Inventory (AFI) was used to measure leadership, strength and low introversion. Pratto et al. [40] Social Dominance Orientation Scale (SDOS) was used to measure social dominance. Rosenberg’s [140] Self-Esteem Scale (RSES) was used to measure self-esteem. Cohen’s [141] Social Network Index (SNI) was used to measure network size and network diversity. In addition to the 21 variables, management position, as an index of leadership role in the workplace was also used as a potential predictor of alpha female status.

### Participants, recruitment and exclusion

A survey of 96 questions designed to collect data on all variables was developed and made available to women aged 18 years of age and older on the website surveymonkey.com. The link to this survey was posted on LinkedIn, Facebook and Twitter, as well as on the websites of various women’s organizations in Canada. Terms of service were adhered to for all social media websites where data were collected. Women who were recruited from malls and universities were given a paper version of the survey to complete.

Of the 512 surveys obtained, 114 participants were excluded from the dataset leaving a total of 398 women as the study population. Participants were excluded if surveys were incomplete or had missing information. The survey results of all 398 participants, a small cross-section of North American society, included career women, housewives, single women, grandmothers, stay at home moms and others. The working description developed from the textual analysis, along with questions designed to assess women’s perceptions of the expression of the alpha female identity in North American/Canadian society were included at the end of the survey. Women were subsequently asked to indicate whether they identified as alpha female or not. Respondents were given the options of “yes, maybe, or no”. Women who responded “yes” were categorized as alpha (N = 94). Women who responded “maybe” or “no”, were categorized as non-alpha (N = 304). For both the alpha and non-alpha female groups, the average age was between 35–37 years, average education level was a bachelor’s degree, and those women who were employed earned an average income of approximately $58,000 annually.

### Measures used in the study

#### Bem Sex Role Inventory (BSRI)–masculine and feminine personality traits

The original BSRI [124] includes 60 dichotomous items divided into 3 subscales—masculinity, femininity, and neutral. Each subscale includes 20 adjectives for subscale that represent typical masculine (Cronbach’s α = 0.86), feminine (Cronbach’s α = 0.86), and 20 neutral traits in Western society. For the purposes of this study, a condensed version of 10 (5 masculine and 5 feminine) items was derived from the original BSRI 60-item scale. These items are representative of the alpha female masculine and feminine themes consistent with the results from the textual analysis. These are represented as BSRI-M (Cronbach’s α = 0.64) and BSRI-F (Cronbach’s α = 0.72). Five (5) neutral items were also included. Respondents were asked to score each item on a 5-point Likert Scale (modified from the original 7-point Likert Scale) from *never* (1) to *always* (5). Means for masculine and feminine categories were calculated to derive corresponding masculinity and femininity scores. Neutral items served as distractors or filler items. Higher masculinity scores indicate higher affiliation with masculine traits, higher feminine scores indicate higher affiliation with feminine traits, equal scores in both masculine and feminine traits indicate androgyny, and low scores in both masculinity and femininity indicate an undifferentiated gender.

Though primarily used to compare masculine and feminine traits in research that includes both men and women [130, 133–134], in the present research, the BSRI is used to examine the expression of masculine and feminine traits in alpha females as well as any difference in these traits between alphas and non-alphas.

#### Collaboration Inventory (CI)—persuasiveness, consensus-building, coalition-building, and networking abilities

The textual analysis revealed persuasiveness, consensus-building, coalition-building, and networking abilities as traits associated with the alpha female. For the purposes of the present research, the Collaboration Inventory (CI) survey instrument was developed and is introduced. Higher scores represent higher self-reported collaboration. All items were answered using a 5-point Likert Scale from *strongly disagree* (1) to *strongly agree* (5). The collaboration mean scores were used as an index of collaboration.

#### Leadership, strength and low introversion (extroversion)–Alpha Female Inventory (AFI)

The subscales of the Alpha Female Inventory (AFI) developed by Ward et al. [11], was used to measure leadership (AFI-L) (Cronbach’s α = 0.82), strength (AFI-S) (Cronbach’s α = 0.72) and extroversion (AFI-LI) (Cronbach’s α = 0.83). The AFI is a 14-item measure of alpha female personality. Items are scored on a 5-point Likert scale from *strongly disagree*-1 to *strongly agree*-5 and summed with higher scores indicating greater levels of leadership, strength, and low introversion (a measure of extroversion). Defined by Ward et al. [11] as “being quiet and withdrawn from social situations” (p. 317), low introversion is considered a proxy measure of extroversion. AFI-LI items are reverse coded where higher scores indicate lower levels of being quiet and withdrawn.

#### Rosenberg Self-Esteem Scale (RSES)–self-esteem

The Rosenberg Self-Esteem Scale (RSES) [140] is a validated measure of self-esteem. Composed of 10 items that assess both positive and negative feelings about the self or “self-worth”, it is the most widely used self-report instrument of confidence and self-esteem [142]. The RSES is unidimensional and items (Cronbach’s α = 0.77–0.88) are scored on a 4-point Likert-type scale ranging from *strongly disagree* (0) to *strongly agree* (3) [142]. The RSES was modified to reflect an additional choice of *neutral* (3) to avoid neutral response bias. Research suggests that because negatively worded items may be interpreted differently by different groups, using the RSES may have limited value [143]. Inclusion of a neutral avoids responses at the extreme ends of the RSES [143]. The result was in a 5-point Likert Scale ranging from *strongly disagree* (1) *to strongly agree* (5). Some questions were also reworded for simplicity and clarity. Items were summed, and higher scores indicated greater self-esteem.

#### Sexual dominance aspects–frequency, dominant role, sexual experience, initiating, and enjoying sex

Questions were designed and included in the survey to assess 5 aspects of sexual dominance which included sex frequency, dominance role, taking initiative in sexual encounters, enjoyment during sex, and sexual experience. Data on alpha and non-alpha sexual preferences (men, women, or both) were also collected and examined.

#### Social Dominance Orientation Scale (SDOS)–social dominance

The Social Dominance Orientation Scale (SDOS) was used to assess preference for group-based hierarchy. The Social Dominance Orientation Scale (SDOS) was used as an index of social dominance with higher SDO scores reflecting higher levels of agreement with the concept of social dominance. The 16-item SDOS (Cronbach’s α = 0.88) uses a 7-point Likert scale of agreement from *very negative* (1) to *very positive* (7) [40]. A modified version of the SDOS was developed and included in the survey instrument. The result was a 12-item instrument scored on a 5-point Likert scale of agreement from *strongly disagree* (1) indicating low SDO, to *strongly agree* (5) indicating high SDO. The purpose was to simplify the measure of the alpha female personality as reflected in the data collected on current definitions and descriptions. A person with low SDO prefers relationships between social groups to be equal and not hierarchical.

#### Life satisfaction and social capital

The present study also included an assessment of life satisfaction. A single question was asked, “Describe the level of satisfaction and fulfillment you feel in your life”. Responses were scored on a 5-point Likert scale (0-none, 1-low, 2-moderate, 3-high, and 4-extremely high). Cohen’s Social Network Index (SNI) (Cronbach’s α = 0.77 [141] was used as a measure social capital. Cohen et al.’s [141] work on social ties and susceptibility to the common cold revealed that individuals with more diverse social networks had greater resistance to upper respiratory illness, even without considering the nature of those social relationships. Network diversity is defined as the number of social roles in which the respondent has regular contact with others. The maximum number of high-contacts is 12 and includes: spouse, parent, child, in-law, close relative, close friend, religious group member, student, employee, neighbor, volunteer, and another group member. Network size is defined as the total number of people with whom the respondent is in regular contact. Both network diversity and network size are based upon having contact at least once every two weeks. Many women in the focus groups indicated that adult grandchildren were very important to their social network. In order to capture the specific value that this may bring to their social networks, a single question, “If you have adult grandchildren how many of them do you see or communicate with (including phone, texting and email) at least once every 2 weeks?”, was added to the original 12-item instrument increasing the maximum number of possible contacts from 12 to 13.

## Ethics statement

The present research, including the method of obtaining informed consent, was approved by the University of Toronto’s Research Ethics Board (Protocol #27117). Informed consent was obtained for each phase of the study from all participants. For the survey portion, informed consent was provided electronically on the surveymonkey.com webpage. The webpage was designed such that participants could not proceed to the survey unless they clicked the “yes” option at the bottom of the consent letter describing the research.

## Statistical analyses

Descriptive statistics including the mean, median and standard deviation were run for all variables. For the analysis of the alpha female, self-identification was used to categorize women as alpha or non-alpha. The differences between the alpha and non-alpha groups were assessed using nonparametric Mann-Whitney U-tests. The Mann-Whitney comparisons were used to identify potential predictor variables of alpha female status. Logistic regression was conducted using ten potential predictor variables that were identified by the Mann-Whitney U comparisons, 1) BSRI-M, 2) Leadership, 3) Strength, 4) Low Introversion, 5) RSES, 6) Life Satisfaction, 7) Sexual Experience, 8) Initiates Sex, 9) Enjoys Sex, and 10) DomRole_Sex. Odds ratio analyses were conducted to examine both the alpha female as a valid form of female identity in Western society, and the likelihood of an alpha female holding a management position. Data on sexual preference were also analyzed to provide insight into the alpha female’s sexuality profile. All statistical tests were conducted using the Number Cruncher Statistical Systems (NCSS) statistical software package [144].

## Results

### Textual analysis

The following themes were revealed by the textual analysis using NVivo [137]. In comparison to non-alpha females, alpha females are described as, 1) more socially dominant, 2) leaders 3) having higher self-esteem, 4) displaying more masculine than feminine personality traits, 5) using collaboration and affiliation strategies to achieve their goals, 6) physically stronger, 7) more extroverted, and 8) more sexually dominant (i.e. more sexually active, play a dominant role in sexual encounters, initiate sex more often, and enjoy sexual intercourse more), than non-alpha women The results of the textual analysis also revealed 21 potential alpha female predictor variables. These included, 1) masculine traits (aggressiveness, ambition, assertiveness, competitiveness, and independence); 2) feminine traits (affectionate, gentle, loyal, sensitive to the needs of others, understanding); 3) neutral traits (conscientious, adaptable, reliable, likeable, tactful); 4) age; 5) education; 6) employment; 7) income; 8) leadership; 9) strength; 10) low introversion; 11) collaboration (persuasiveness, consensus-building, coalition-building, and networking abilities); 12) social dominance; 13) self-esteem; 14) network size; 15) network diversity; 16) life satisfaction; 17) sex frequency; 18) sexual experience; 19) initiates sex; 20) enjoys sex; and 21) plays a dominant role in sexual encounters. These results allowed for the development of the following working definition of the alpha female/woman:

*“The alpha female is a confident leader who is socially and sexually dominant over others*. *She is physically strong*, *more sexually active*, *and extroverted*, *and her personality is more masculine than feminine*. *She believes that men and women are equal and uses collaboration and affiliation strategies to achieve her goals*.*”*

### Assessing the social construction of the alpha female

To assess social construction of the alpha female identity, levels of association for 3 specific questions were calculated (see Table 1). To test whether women occupy the alpha female identity, odds ratio analyses were conducted (see Table 2). Self-identified alpha females were 2.4 times more likely to have heard of the term alpha female/woman than non-alpha females. Of all 398 women, 91% of alpha females and 82% of non-alpha females had heard of the term. Combined, 84% off all women had heard of the terms “alpha female” or “alpha woman”. Alpha females were 8.6 times more likely to agree that the alpha female is more than just a construct of popular media and exists as a true identity in Canadian society than non-alpha females. Of all 398 women, 94% of alpha and 63% of non-alpha women agreed with this statement. Alpha females were 7.5 times more likely to agree that the alpha female is a positive form of female identity than non-alpha females. Of all 398 women, 85% of alpha and 43% of non-alpha women agreed that the alpha female is positive form of female identity. Odds ratio analysis was also used to test the association between alpha status and leadership position in the workplace as measured by management level (Table 2). Interestingly, alpha and non-alpha females were equally likely to occupy management positions and of the 59 alpha women who were employed, 81% did not. None of these associations as determined by 95% CI were statistically significant.

**Table 1:** Alpha female identity occupation.

1. Have you heard of the alpha woman? (N = 398)					
Yes 84%					
No 16%					
2. The alpha female is a true form of female identity in Canadian/Western Society (N = 398)					
Yes 70%					
No 30%					
3. The alpha woman is a positive female identity (N = 398)					
Yes 55%					
Neutral 38%					
No 7%					

**Table 2:** Associations between self-identified alpha and non-alpha females for identity occupation and management position.

1. Have heard of the alpha female/woman					
	Yes	No	Odds Ratio	CI (95%)	
Alpha	86	8			
Non-Alpha	249	55	2.37	1.09–5.19	
2. The alpha female is a true form of female identity in Canadian/Western society					
	Yes	No	Odds Ratio	CI (95%)	
Alpha	88	6			
Non-Alpha	192	112	8.56	3.62–20.20	
3. The alpha woman is a positive female identity					
	Yes	No	Odds Ratio	CI (95%)	
Alpha	80	14			
Non-Alpha	131	173	7.55	4.09–13.91	
4. Management					
	Yes	No	Odds Ratio	CI (95%)	
Alpha	131	173			
Non-Alpha	249	55	0.99	0.47–2.08	

### Expression of the alpha female identity

To test the expression of the alpha female identity univariate analyses were conducted (Table 3). Correlation (Table 4) and multinomial logistic regression analyses (Table 5) were also conducted to assess the relationships among variables. Positive and highly significant differences in mean scores between alpha (N = 94) and non-alpha (N = 304) females were found for 10 variables (Table 3) including, BSRI-M, Leadership, Strength, Low Introversion, RSES, Life Satisfaction, Sexual Experience, Initiates Sex, Enjoys Sex. Alpha females exhibited a higher average score for playing a dominant role in sexual encounters (DomRole_Sex), though this difference did not quite reach significance. Small but non-significant differences in Age, BSRI-N, Employment, Income, Collaboration, SDO, and Network Size were found with alpha females scoring slightly higher than non-alphas. Alpha females scored slightly lower in Education than non-alphas though this difference was also non-significant. There was no difference in BSRI-F, Network Diversity, and Sex Frequency.

**Table 3:** Result from univariate analyses for the self-identified alpha and non-alpha female groups.

		Alpha			Non-Alpha		
Variable	N	Mean	SD	N	Mean	SD	P
**1.Masculine Traits (BSRI-M)**	94	3.938	0.438	304	3.536	0.514	**<0.0001**
2.Feminine Traits (BSRI-F)	94	4.153	0.457	304	4.105	0.507	0.421
3.Neutral Traits (BSRI-N)	94	4.257	0.389	304	4.166	0.463	0.065
4.Age Category	94	3.745	1.182	304	3.572	1.149	0.235
5.Education	94	3.213	1.252	304	3.398	1.026	0.124
6.Employment	94	3.249	0.761	304	3.046	0.739	0.175
7.Income	94	3.638	1.789	304	3.579	1.702	0.842
**8.Leadership**	94	20.011	3.416	304	18.516	3.647	**<0.001**
**9.Strength**	94	16.883	2.29	304	15.812	2.559	**<0.001**
**10.Low Introversion**	94	14.575	2.546	304	12.707	3.335	**<0.0001**
11.Collaboration	94	16.101	2.546	304	15.684	2.488	0.156
12.Social Dominance (SDO)	94	27.702	6.596	304	26.697	6.346	0.264
**13.Self-Esteem (RSES)**	94	39.68	6.045	304	37.72	6.38	**<0.01**
14.Network Size (SNI)	94	23.511	9.667	304	22.103	8.816	0.26
15.Network Diversity (SNI)	94	6.617	1.913	304	6.625	1.913	0.927
**16.Life Satisfaction**	94	2.787	0.746	304	2.546	0.811	**<0.01**
17.Sex Frequency	91	1.231	1.146	288	1.226	1.049	0.767
**18.Sexual Experience**	90	2	1.161	290	1.624	1.022	**<0.01**
**19.Initiates Sex**	90	2.8	0.902	290	2.541	0.836	**<0.05**
**20.Enjoys Sex**	89	4.36	0.843	291	4.041	1.082	**<0.05**
**21.DomRole_sex**	90	2.455	0.85	290	2.283	0.81	0.057

**Table 4:** Nonparametric spearman correlations among variables used in the study (N = 398).

	Age Cat.	Education	Leadership	Strength	Low Introversion	Collab	Social Dominance (SDO)	Self-Esteem (RSES)	Network Size (SNI)	Network Diversity (SNI)	Emp	Inc	Sex Freq	Sexual Exp.	Masc. Traits (BSRI-M)	Fem. Traits (BSRI-F)	Neut. Traits (BSRI-N)	Initiates Sex	Dom Role_sex	Life Sat.	Enjoys Sex
Age Cat.	1.000																				
Education	-0.020	1.000																			
Leadership	0.162	0.245***	1.000																		
Strength	0.101	-0.072	0.229***	1.000																	
Low Introversion	0.146	-0.082	0.451***	0.423***	1.000																
Collab	0.147	0.021	0.223***	0.129	0.215	1.000															
Social Dominance (SDO)	0.156	0.111	0.255***	0.148	0.415***	-0.024	1.000														
Self-Esteem (RSES)	0.335***	0.194	0.452***	0.299	0.466***	0.195	0.366***	1.000													
Network Size (SNI)	0.067	-0.030	0.158	0.200	0.204	0.210	-0.006	0.209	1.000												
Network Diversity (SNI)	0.095	0.073	0.159	0.137	0.197	0.154	0.082	0.216	0.653***	1.000											
Emp	0.381***	-0.123	0.230	0.138	0.278	0.083	0.210	0.226	0.019	0.056	1.000										
Inc	0.337***	0.112	0.332***	0.157	0.338***	0.099	0.573***	0.573***	0.063	0.119	0.177	1.000									
Sex Freq	-0.181	-0.005	-0.012	0.113	0.059	0.165	-0.010	0.048	0.114	0.078	-0.036	-0.014	1.000								
Sexual Exp.	0.196	-0.031	0.107	0.174	0.120	0.058	0.078	0.043	-0.066	-0.100	0.110	0.141	0.006	1.000							
Masc. Traits (BSRI-M)	-0.047	-0.060	0.293***	0.517***	0.531***	0.046	0.210***	0.268***	0.117	0.071	0.094	0.140	0.049	0.179	1.000						
Fem. Traits (BSRI-F)	0.023	-0.069	0.070	0.142	0.131	0.207***	-0.112	0.188	0.182	0.191	0.035	-0.059	0.116	-0.098	0.081	1.000					
Neut. Traits (BSRI-N)	0.112	-0.035	0.173	0.267***	0.241***	0.247***	0.004	0.359***	0.208	0.168	0.033	0.064	0.109	-0.045	0.215	0.556	1.000				
Initiates Sex	-0.092	0.035	0.063	0.150	0.096	0.078	-0.015	0.048	0.075	0.036	0.081	0.002	0.280***	0.103	0.113	0.129	0.142	1.000			
Dom Role_sex	-0.012	0.040	0.085	0.126	0.045	0.042	-0.013	0.016	0.086	0.018	0.041	-0.013	0.203	0.075	0.130	0.020	0.054	0.511***	1.000		
Life Sat.	0.124	0.024	0.158	0.218***	0.139	0.111	0.009	0.451***	0.271	0.190	0.113	0.089	0.171	-0.083	0.173	0.255	0.304	0.108	0.128	1.000	
Enjoys Sex	0.010	-0.056	0.093	0.248***	0.164	0.119	0.022	0.182	0.093	0.014	0.056	0.043	0.298***	0.130	0.171	0.198	0.216	0.439***	0.320***	0.214	1.000

***P<0.0001

**Table 5:** Results from the logistic regression analysis (N = 380).

Predictor	Regression Coefficient	Standard Error	Wald Z-value	P-value
Constant	-9.011	1.495	-6.027	0
**BSRI-M**	**1.4**	**0.355**	**3.941**	**<0.0001**
Leadership	0.045	0.043	1.041	0.297
Low Introversion	0.07	0.058	1.216	0.224
**Life Satisfaction**	0.359	0.197	1.822	**0.068**
RSES	-0.022	0.029	-0.758	0.449
**Sexual Exp.**	**0.238**	**0.128**	**1.857**	**0.063**
Enjoys sex	0.053	0.165	0.323	0.747
Initiate Sex	0.204	0.19	1.075	0.282
Dom Rol_Sex	-0.019	0.189	-0.098	0.922
Strength	-0.03	0.069	-0.432	0.449

The results from the nonparametric correlation analysis revealed several positive and highly significant relationships (Table 4). Self-esteem was correlated with life satisfaction, income, social dominance, BSRI-M, leadership and low introversion. Social dominance was highly and positively correlated with income, BSRI-M, leadership and low introversion. Leadership, strength and low introversion were positively correlated with BSRI-M (masculine traits). Leadership and low introversion were positively correlated with income. Other positive and highly significant correlations included BSRI-N (neutral traits) with RSES (self-esteem) and life satisfaction as well as age and employment status. Life satisfaction was correlated with strength. Sex frequency was positively and highly correlated with initiating and enjoying sex, as taking a lead or dominant role in sexual encounters was with initiating sex. Social capital (network diversity and network size) were not correlated with any other variables.

BSRI-M was the only significant predictor of alpha female identity as identified by the multiple logistic regression model (Table 5), with sexual experience and life satisfaction approaching statistical significance. For both the alpha female and non-alpha female groups, mean BSRI-F scores were higher than mean BSRI-M scores (Table 3). The model correctly classified only 23% of alpha females (Table 6). There was little difference between alpha and non-alpha females with respect to sexual preference (see Table 7).

**Table 6:** Classification table.

	Estimated		
Actual	Non-Alpha	Alpha	Total
**Non-Alpha**	276	12	95.8%
**Alpha**	70	19	21.3%
**Total**	346	31	

**Table 7:** Sexual preference: Alpha and non-alpha females.

Preference	Non-Alpha	%	Alpha	%	Total
Men	249	86%	80	88%	329
Women	24	8%	7	8%	31
Both	18	6%	4	4%	22
Total	291		91		382

## Discussion

The present study investigated the alpha female as a form of social identity in a small non-random sample of women in North America (N = 398). The association between self-identified alpha females and measures of sexuality, and management, as an index of leadership position in the workplace were also examined. Based on the textual analysis it was expected that the alpha female would be considered a widely recognized and positive social construct of female identity that privileges masculine traits akin to the alpha male. Two hypotheses were developed to test the two competing conceptualizations of the alpha female identity in Western society, 1) The Alpha Female–Masculine (AFM) Hypothesis and 2) The Alpha Female-Feminine Hypothesis (AFF). Under the Alpha Female-Masculine Hypothesis, it was predicted that alpha females would be more socially and sexually dominant, more likely to occupy a management position in the workplace, be a leader, be stronger, have higher self-esteem, be less introverted than non-alpha females, and possess more masculine than feminine traits. Under the Alpha Female-Feminine Hypothesis (AFF) it was predicted that compared to non-alpha females, alpha females would be more collaborative and possess more feminine than masculine traits.

The results of the present study revealed that for the present sample of North American women, masculine traits (BSRI-M) was the only statistically significant predictor of alpha status. The measure of masculine traits was followed closely by life satisfaction and sexual experience as predictors of alpha status. That is, male personality traits which included being aggressive, ambitious, assertive, competitive and independent, are the only significant predictors of, and contributors to the alpha female identity given scores for all other variables. However, this result did not negate identification with feminine traits. Interestingly, both alpha and non-alpha women scored equally with the feminine traits (BSRI-F) of being affectionate, gentle, loyal, understanding, and sensitive to the needs of others, and both groups identified more with feminine traits than masculine traits. According to Bem [125], individuals who score higher on feminine characteristics than masculine ones, are categorized “feminine”, suggesting that alpha females and non-alpha females can be classified as “feminine”. Most women regardless of self-identified alpha status reported the “alpha female” as a positive, and valid or true construct of female identity.

Collaboration as reflected in coalition and consensus building, networking and persuasiveness, were also equally possessed by both alpha and non-alpha females. Both alpha and non-alpha women had low Social Dominance Orientation (SDO) indicating that regardless of alpha self-identification women in the present study preferred relations between social groups to be more equal rather than hierarchical. Alpha females also reported being physically and mentally stronger, having higher self-esteem, greater life satisfaction, and being less introverted than non-alpha women. Though alpha woman reported leadership as being an important aspect to their identity, they did not necessarily hold a leadership position in the workplace. Aspects of sexual dominance though higher in alpha women were not predictors of alpha status. The results support the Alpha Female-Masculine (AFM) hypothesis however, not at the expense of feminine traits. Being more “male” does not mean being less “female”.

The results of the present study are consistent with previous research which has demonstrated that alpha females possess masculine traits [8, 10–13, 38]. It is also consistent with research that links these traits with leadership, strength and extroversion [11]. However, the results also challenge previous research that identifies social dominance and aspects sexual dominance as key traits of the alpha female [38]. For example, there was no statistically significant difference in Social Dominance Orientation (SDO) between alphas and non-alphas. Additionally, although there were statistically significant differences for specific aspects of sexual dominance (sex frequency, enjoys sex, initiates sex, and playing a dominant role in sexual encounters) with alpha females scoring higher than non-alphas, neither social dominance orientation nor any of these aspects of sexual dominance were found to be predictors of the alpha female identity in the logistic regression model. Furthermore, aspects sexual dominance and social dominance were not correlated. Given the academic and popular literature that state otherwise, these results in particular were not expected. What could explain why alpha females do not exhibit greater social dominance despite research that has demonstrated it to be one of the hallmark traits of an alpha individual?

With respect to sexual behavior, the results revealed that although alpha and non-alpha females do not differ in terms of the frequency of sex, they are different when it comes to sexual experience (number of partners), initiating and enjoying sexual intercourse with alpha females reporting higher levels in all. Both alpha and non-alphas however, reported that between “sometimes” and “half the time”, they initiate and play a dominant role in sexual encounters. Both alpha and non-alpha females exhibit a mean score for sexual experience that suggest fewer than what they would consider to be an average number of partners. The results of the present research challenge assumptions about the alpha female as promiscuous and more sexually dominant than non-alpha females. For example, recent research has shown that alpha males and females prefer to submit rather than dominate in the bedroom; that they prefer a role reversal [37]. Thus, the alpha female, sometimes dominant in one social domain, may prefer not so to be in another. These discrepancies in relating the concept of social dominance in primates and other animals to humans and how hierarchies are subsequently formed and maintained, render as questionable, the trait of social dominance, and by extension sexual dominance, as predictors of alpha female status. Thus, contrary to academic and popular discourses in the animal, primate and human research on the alpha personality, for the human alpha female, perhaps social and sexual dominance are understood and perceived differently, or do not factor at all.

The results also add to recent research on leadership which demonstrates that it is advantageous for both male and female leaders to have masculine and feminine attributes [145]. They also challenge the often-assumed inextricable link, and interchangeable use, of the concepts of leadership and the alpha female. The analysis revealed that although there was a highly significant difference in mean leadership scores between alpha and non-alpha females, with alpha females scoring higher, alpha and non-alpha females were equally likely to hold a leadership/management position at work. These results therefore, not only challenge the common assumption that alpha females are leaders, they also challenge previous leadership research that present the alpha female as a “special kind of leader” [10].

### Limitations and future research

The results of the present research are subject to several important limitations. Firstly, the present study relies on self-identification as the method of identifying alpha and non-alpha women and does not include data on how those women would be identified by their peers. The degree to which individuals and groups are perceived by others or non-group members represents another dimension [136]. As such, though some women may self-identify as alpha female, this identification may or may not align with the perceptions and beliefs of others [146]. Future research that considers the opinions and perceptions of non-alpha women and men, of particular self-identified alpha women may also provide insight into the social construction of the alpha female identity.

As data were collected through the survey, focus groups and interviews, self-identified alpha females were not observed in their natural environments limiting the insight into the potential variation in the expression of the identity. Humans have complex social lives and operate in varying contexts, belong to different social groups and hierarchies, and perform a variety of social roles [32]. Within this context, self-identifying as alpha female may not necessarily mean that she is all alpha, all of the time. For example, in two studies conducted with approximately 900 college students (men and women), Hawley and Hensley [37] found that when it comes to connecting on a sexual level with dominant or alpha males, women who identified as alpha, preferred forceful submission fantasies more than women who identified as subordinate [37]. Similarly, others have found that although alpha females like to stay in control in the bedroom, they are willing to relinquish this control to men when it suits them [15, 98]. Thus, the alpha female does not always choose to be alpha in every context. Further, people tend to value those hierarchies in which they are ranked the highest. For example, a person who works in the mailroom of their company and is the top baseball player on the company team, may derive more self-esteem from the latter hierarchy [32]. Additionally, depending upon the context, people also tend to alter the psychological meaning of a rank [32]. For example, a novice runner who completes a marathon would be more pleased with themselves than a person who was expecting to win but placed 5^th^ [32]. Future ethnographic research focused on the daily lived experiences and the various contexts of the alpha female may provide greater insight into the potential fluidity and variation in the expression of the alpha female identity.

Although measures of sex frequency, sex enjoyment, dominant role in sexual encounters, initiation of sex, and sexual experience were included in the present study, data on the type of sexual activity performed were not collected. To date, research on the type of sexual activity with respect to the alpha female identity has not been undertaken. There has been some research however, which has demonstrated that some forms of sexual activity positively contribute to life satisfaction (i.e. penile-vaginal penetration for women is linked to lower life stress than oral sex, or masturbation [147, 148]. Further research into which forms of sexual activity alpha females perform may provide greater insight into the alpha female’s sexual profile.

The present study is also limited by non-random sampling, and therefore may include some bias, the nature of which is unknown. It is also potentially limited by small sample sizes for both the non-alpha and alpha sampling groups. It is therefore possible the results do not accurately reflect the differences between these groups of women in North America. The non-random small sample also limits the potential to generalize to a larger population beyond the scope of the present study.

Use of the BSRI also poses a limitation. In some cases, the BSRI has been shown not to be a valid measurement of psychological androgyny due to localized constructions of masculinity and femininity [149]. Thus, the BSRI may not accurately reflect gender expression in other cultures and societies for example, matrilineal societies such as the Minangkabau of Indonesia [150] and the Mosuo of China [151]. Examining the alpha female construct in such societies may require a modification of the current BSRI to accommodate for such cross-cultural differences.

Finally, though data on the ethnicity of women was collected it’s influence on social rank and potential impact on social hierarchy was not part of scope of the present research. Data on sexual orientation was not collected or examined as a potential influencer of social hierarchy. The present research examined hierarchy strictly in the sense of being exhibited through social dominance. An examination of how social rank is expressed through such aspects and how they may or may not intersect with the identity itself may provide more context to the alpha female as a value-laden identity.

## Conclusions

The present research contributes to and has direct implications for future leadership and alpha-leadership research. In the leadership literature, although the term “alpha” has become synonymous with the term “leader” [10, 11, 14, 88, 89] there is confusion as to what “alpha leadership” actually means. Those at “the top” of the business hierarchy or organizational chart are considered the leaders. “Alpha leadership” is a term often used to describe a leadership style for those holding top positions in organizations such as CEOs and senior management [152]. While some claim leadership for these individuals is social dominance [38], others contend that such leadership has little to do with it [152]. Similarly, when it comes to identifying alpha males and females there seems to be confusion. According to [88], it is easy to spot an alpha male in the workplace, but spotting an alpha female is more challenging. The authors contend that this occurs because people are more “confused” about the alpha female when it comes to her traits [88]. They argue that different alpha females may possess some but not all of the traits possessed by alpha males [88]. Irrespective of the confusion, leaders in organizations and other leaders such as politicians, exercise influence over others; play a lead role in goal-setting, goal achievement, and the development of a group or organization; and are regarded as the leader by other members of the group [17]. The present research therefore also serves as a framework within which to also evaluate the alpha male within this context. Further, a comparison of the alpha male and female will provide invaluable and quantifiable insight into the notion of alpha leadership. The results of such a study would have a significant impact on how leadership is viewed in the workplace and what traits/characteristics employers should look for when hiring for specific leadership positions.

Though previous research on the alpha female has been used to explain the influence of the shift in women’s roles, it is largely based on assumptions regarding the relationship between leadership and the alpha female and the validity of the alpha female as a universal identity/category in society. It is argued that in an effort to engage in the gender-equality discourse, such assumptions have contributed to the notion that the alpha female is the female representation of her male counterpart, the alpha male. In turn, this has added to the discourse that leadership and being alpha female are inextricably linked. Whether women identify as alpha female or not, or whether women consider the alpha female as a valid identity at all, has not been considered previously. The present research is thus significant as it is the first to use self-identification to identify alpha females as opposed to identification through predetermined and assumed alpha female behavior traits, including leadership. Additionally, the present research is the first to validate the alpha female as a valid and socially constructed positive female identity in Western society and to identify specific male personality traits (aggressive, ambitious, assertive, competitive, and independent) as predictors of alpha female status. In asking women themselves what they think about the alpha female what has come to light is that some descriptions of the alpha female hold true while others do not. In particular, the absence of social dominance as a predictor of alpha female status in the study sample a major finding, warrants a refocus of our understanding of the alpha female as a dominant individual in society. The findings also add to the leadership research, namely that female leaders in the workplace (i.e. women in management and senior management positions) do not necessarily identify as alpha. For those that do, there are other traits including non-masculine traits to consider. Consideration of such traits may have implications for organizations who rely on masculine gender stereotype alpha traits as indicators of leadership/management quality and potentiality.

Contrary to popular narratives, for the study population, the alpha female does not necessarily have sex more frequently than other women though she is more experienced and enjoys sex more. She does not necessarily make more money or is more educated than other women, and she does not necessarily hold a senior position in her workplace. She reports being strong and extroverted and being aggressive, ambitious, assertive, competitive, and independent however, not at the expense of being affectionate, gentle, loyal, sensitive to the needs of others, and understanding.

The results of the present study revealed that the human alpha female is far more complex when its social construction and measurable expression of the identity are included in the discourse. This suggests that current understandings, popularized notions, and academic research on the alpha female are incomplete, and as such, have ramifications for research that seek to identify or categorize women to a specific female identity.

## Supporting information

S1 Fig Google Ngram viewer for the search term “alpha woman”. (TIF)

S2 Fig Google Ngram viewer for the search term “alpha female”. (TIF)

S1 File Search terms for data queries. (DOCX)

S2 File Social dominance orientation scale. (DOCX)

S3 File Rosenberg self esteem scale. (DOCX)

S4 File BSRI-M, BSRI-F, and BSRI-N. (DOCX)

S5 File Collaboration Index (CI). (DOCX)

S6 File Alpha female sexuality profile. (DOCX)

S7 File Alpha Female Inventory (AFI). (DOCX)

S8 File N = 398 Dataset. (PDF)
