# Supplementary figures and images for: Masculinity, femininity, and leadership: Taking a closer look at the alpha female
Source: PLoS One. 2019 Apr 12;14(4):e0215181. doi: 10.1371/journal.pone.0215181 (PMC6461231; doi:10.1371/journal.pone.0215181)

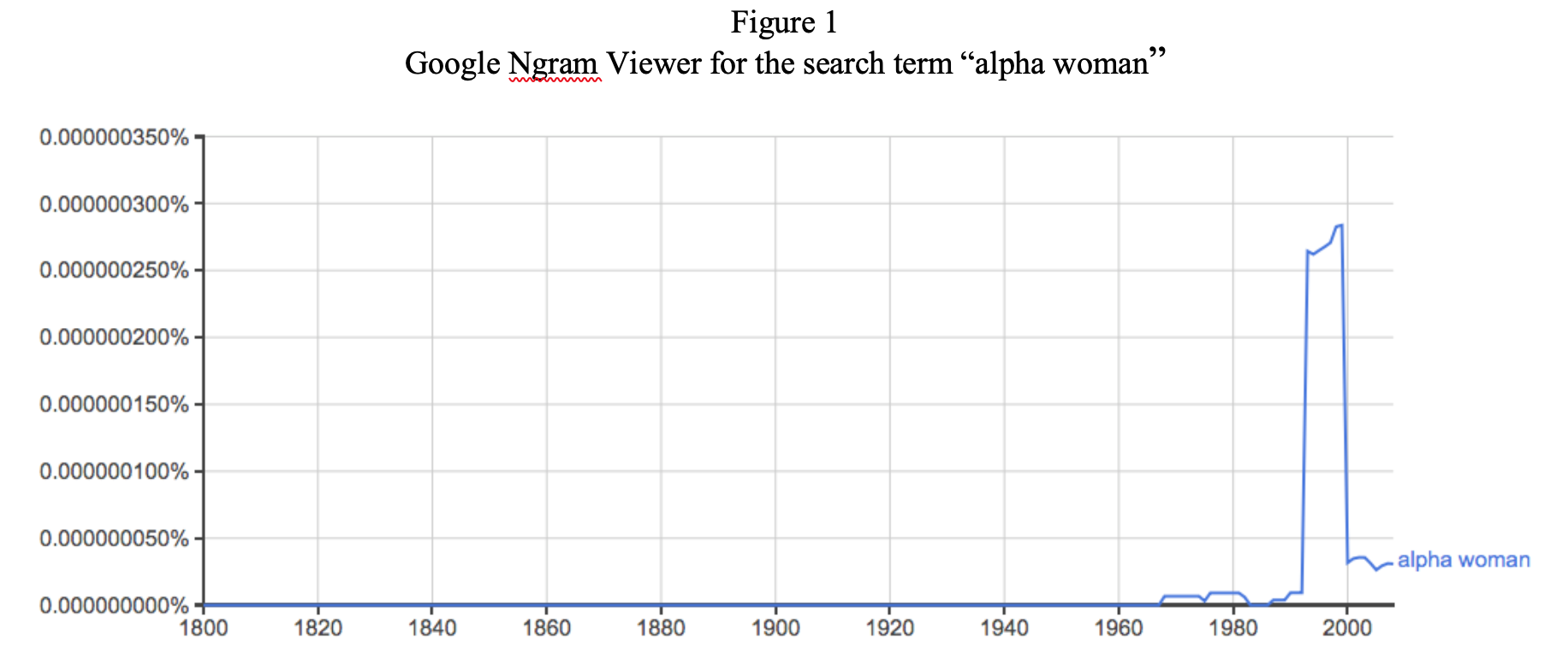

Supplement: S1 Fig — (TIF) [file pone.0215181.s001.tif]

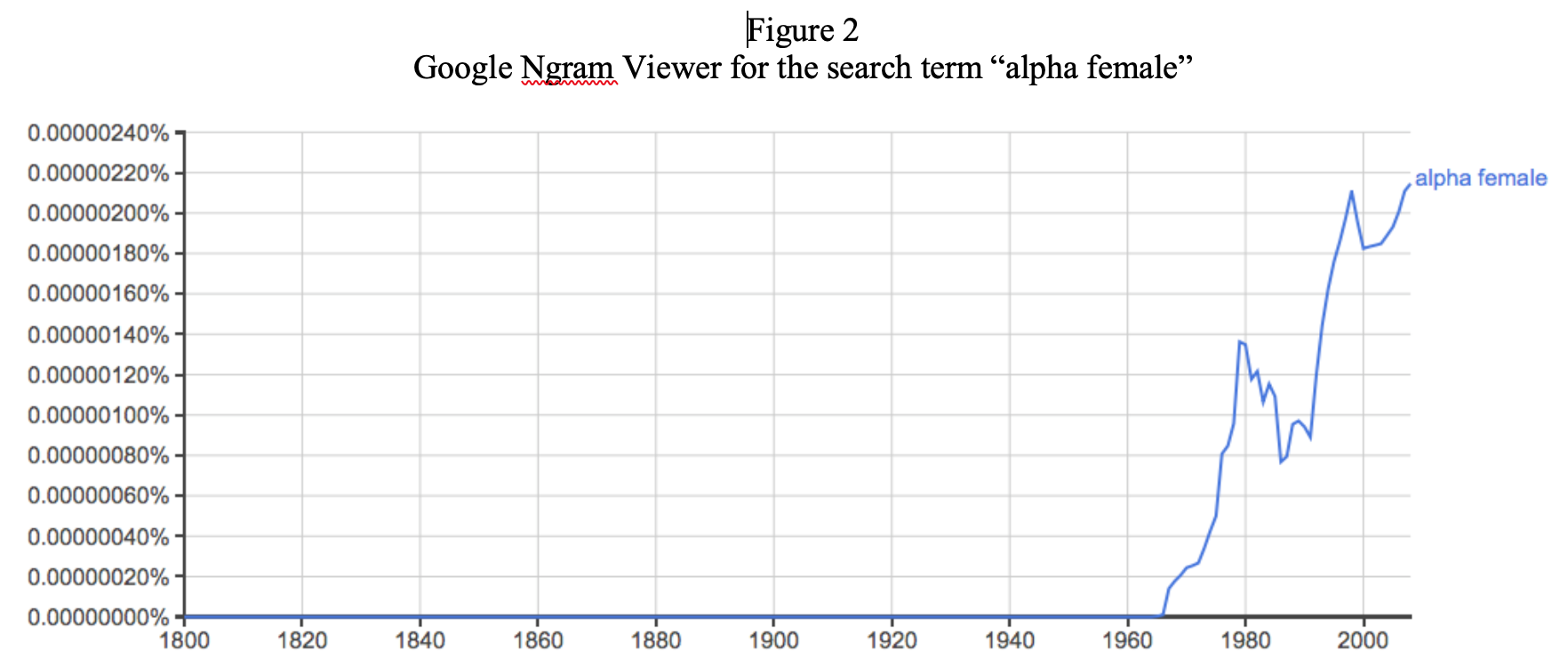

Supplement: S2 Fig — (TIF) [file pone.0215181.s002.tif]
