# Supplementary material for: Masculinity, femininity, and leadership: Taking a closer look at the alpha female
Source: PLoS One. 2019 Apr 12;14(4):e0215181. doi: 10.1371/journal.pone.0215181 (PMC6461231; doi:10.1371/journal.pone.0215181)
Supplement: S1 File — (DOCX) [file pone.0215181.s003.docx]

S1 Appendix

Search Terms for Data Queries

alpha female, alpha woman, alpha women alpha girl, alpha man, alpha male, alpha men, femininity, masculinity, masculine traits, male traits, feminine traits, female traits, education, income, age, management position, leadership, strength, extroversion, low introversion, collaboration, social dominance, social support, social network, communal, cooperative, satisfaction with life, promiscuity, sex frequency, sexual dominance, dominant role in sexual encounters, sexual experience, sex partners, self-esteem, confidence, aggression, assertiveness, independence, competitiveness, independent, affectionate, caring, sensitive to the needs of others, loyal, understanding, alpha female the same/different as the alpha male, success, leadership position, leadership role, women’s leadership, female leadership, male leadership, hierarchy, social hierarchy, alpha female identity
