# Supplementary material for: Masculinity, femininity, and leadership: Taking a closer look at the alpha female
Source: PLoS One. 2019 Apr 12;14(4):e0215181. doi: 10.1371/journal.pone.0215181 (PMC6461231; doi:10.1371/journal.pone.0215181)
Supplement: S2 File — (DOCX) [file pone.0215181.s004.docx]

S2 Appendix

Social Dominance Orientation Scale (Pratto et al., 1994)

1. We would have fewer problems if we treated people more equally
2. Some groups of people are simply inferior to others
3. In getting what you want it is sometimes necessary to use force against others
4. Hierarchies exist in society. It’s just a fact of life, and that is ok
5. We should do what we can to equalize conditions for different groups
6. Some people are just more deserving than others
7. We should have increased social equality
8. It is ok if some groups have more of a chance in life than others
9. We should strive to make incomes as equal as possible
10. To get ahead in life it is sometimes necessary to step on others
11. Group equality should be our ideal
12. No one group should be dominant in society

Note: All items were scored on a 5-point Likert Scale from *strongly disagree* (1) to *strongly agree* (5). Items 2, 3, 4, 6, 8, and 10 were reverse coded.
