# Supplementary material for: Masculinity, femininity, and leadership: Taking a closer look at the alpha female
Source: PLoS One. 2019 Apr 12;14(4):e0215181. doi: 10.1371/journal.pone.0215181 (PMC6461231; doi:10.1371/journal.pone.0215181)
Supplement: S3 File — (DOCX) [file pone.0215181.s005.docx]

S3 Appendix

Rosenberg Self Esteem Scale (Rosenberg, 1965)

1. I am satisfied with myself
2. Sometimes I think I am no good at all
3. I have many good qualities
4. I am able to do things as well as most other people
5. I don’t feel I have a lot to be proud of
6. Sometimes I feel useless
7. I feel that I am a person of worth, at least on an equal plane with others
8. I wish I could have more respect for myself
9. I often feel like a failure
10. I take a positive attitude toward myself

Note: All items were scored on a 5-point Likert Scale from *strongly disagree* (1) to *strongly agree* (5). Items 2, 5, 6, 8, and 9 were reverse coded.
