# Supplementary material for: Masculinity, femininity, and leadership: Taking a closer look at the alpha female
Source: PLoS One. 2019 Apr 12;14(4):e0215181. doi: 10.1371/journal.pone.0215181 (PMC6461231; doi:10.1371/journal.pone.0215181)
Supplement: S4 File — (DOCX) [file pone.0215181.s006.docx]

S4 Appendix

BSRI-M, BSRI-F, & BSRI-N (Bem, 1974)

BSRI-M

1. Aggressive
2. Ambitious
3. Assertive
4. Competitive
5. Independent

BSRI-F

1. Affectionate
2. Gentle
3. Loyal
4. Sensitive to the needs of others
5. Understanding

BSRI-N

1. Conscientious
2. Adaptable
3. Reliable
4. Likeable
5. Tactful

Note: All items were scored on a 5-point Likert Scale from *never* (1) to *always* (5)
