# Supplementary material for: Masculinity, femininity, and leadership: Taking a closer look at the alpha female
Source: PLoS One. 2019 Apr 12;14(4):e0215181. doi: 10.1371/journal.pone.0215181 (PMC6461231; doi:10.1371/journal.pone.0215181)
Supplement: S5 File — (DOCX) [file pone.0215181.s007.docx]

S5 Appendix

Collaboration Index (CI) (Sumra, 2019)

1. Q23-MSPO I typically prefer to be persuasive rather than forceful.
2. Q24-MSCONO Instead of asserting my opinion, I prefer to build consensus
3. Q25-MSCOAO In the face of adversity I prefer coalition building rather than handling it on my own
4. Q26-MSAFFO I am good at making connections with people in new social settings**.**
