# Supplementary material for: Masculinity, femininity, and leadership: Taking a closer look at the alpha female
Source: PLoS One. 2019 Apr 12;14(4):e0215181. doi: 10.1371/journal.pone.0215181 (PMC6461231; doi:10.1371/journal.pone.0215181)
Supplement: S6 File — (DOCX) [file pone.0215181.s008.docx]

S6 Appendix

Alpha Female Sexuality Profile (Sumra, 2019)

1. In an average month how many times do you engage in sexual intercourse?

None, 1-5, 6-10, 10-15, more than 15 times

1. What is your sexual preference?

men, women, both

1. How often do you initiate sex?

never, sometimes, half the time, most of the time

1. How often do you play a lead or dominant role during your sexual encounters?

never, sometimes, half the time, most of the time, all the time

1. On a 5-point scale how much do you enjoy sexual intercourse?

1=not at all and 5=very much

1. Please select your level of sexual experience (different partners).

very low, low, what I think is average, high
