# Supplementary material for: Masculinity, femininity, and leadership: Taking a closer look at the alpha female
Source: PLoS One. 2019 Apr 12;14(4):e0215181. doi: 10.1371/journal.pone.0215181 (PMC6461231; doi:10.1371/journal.pone.0215181)
Supplement: S7 File — (DOCX) [file pone.0215181.s009.docx]

S7 Appendix

Alpha Female Inventory (AFI) – (Ward et al., 2010)

AFI-L

1. I like to lead group projects
2. My friends know me as the leader
3. I am a dominant force in my areas of interest
4. I am assertive in what I want and believe
5. I am destined to be a leader
6. I look forward to challenges

.

AFI-S

1. I consider myself tough
2. I am physically and mentally stronger than most women I know
3. I am just a girl, I don’t consider myself that strong. MODIFIED “As a woman, I don't consider myself that strong compared to men”
4. I enjoy athletics and physical activity

AFI-LI

1. I consider myself rather shy
2. In social settings, I am usually quiet.
3. I consider myself to be more introverted – MODIFIED “I CONSIDER MYSELF MORE INTROVERTED”
4. I’d rather be behind the scenes as opposed to the forefront

Note: All items were scored on a 5-point Likert Scale from *strongly disagree* (1) to *strongly agree* (5). Items 9, 11, 12, 13 and 14 were reverse coded.
