# Supplementary material for: Masculinity, femininity, and leadership: Taking a closer look at the alpha female
Source: PLoS One. 2019 Apr 12;14(4):e0215181. doi: 10.1371/journal.pone.0215181 (PMC6461231; doi:10.1371/journal.pone.0215181)
Supplement: S8 File — (PDF) [file pone.0215181.s010.pdf]

S8 DATA N=398

AGE CAT	EDU CAT	EMP CAT	INCOME CAT	MGMT STATUS	SELF A_NA	LEADERSHIP	STRENGTH	LOW INTROV	COLLAB	SDO Sum
3	3	3	7	0	2	20	19	14	19	21
4	3	2	7	4	1	27	18	18	19	25
3	3	3	2	3	2	21	15	17	15	26
5	6	3	7	3	2	26	16	18	20	40
6	3	4	3	4	1	18	16	13	19	22
4	3	3	3	2	1	21	16	14	16	28
6	1	4	3	0	1	17	17	14	20	16
3	4	3	3	3	1	14	20	13	16	21
4	3	4	3	0	2	19	20	14	18	24
4	5	3	7	4	1	25	14	15	15	35
4	2	4	3	4	1	23	16	16	19	26
6	2	4	3	4	1	24	16	18	20	22
2	4	2	2	3	1	16	14	14	20	25
6	2	4	7	4	1	13	12	16	16	29
3	3	4	5	4	2	24	16	17	13	31
2	5	3	6	2	1	24	18	15	16	25
3	3	3	5	1	2	21	19	16	19	22
3	3	3	7	3	1	20	17	15	18	40
2	2	3	3	2	1	18	20	16	18	22
3	6	4	2	4	1	24	15	15	15	32
3	3	4	4	2	1	23	19	16	13	23
2	3	3	2	1	1	18	19	14	19	24
4	3	3	3	4	2	26	14	18	15	41
3	4	3	7	3	1	24	13	15	17	43
4	4	3	4	2	1	18	15	16	16	21
3	6	3	4	3	2	22	17	17	18	27
3	3	4	3	0	1	18	16	6	12	27
4	4	4	2	0	2	23	19	14	20	21
4	2	3	3	3	1	14	16	16	18	34
4	4	4	1	1	1	17	17	11	17	20
4	6	4	1	0	2	22	20	14	18	27
4	4	3	7	4	1	20	17	17	17	36
3	2	3	4	3	1	20	14	15	14	31
3	3	4	6	4	2	25	18	18	13	36
4	4	4	7	4	2	16	17	18	13	40
5	3	3	4	3	2	23	20	14	17	28
2	4	2	2	2	1	24	15	11	17	27
3	4	4	2	0	2	23	17	15	11	26
4	2	4	3	4	2	22	13	19	15	29
2	4	1	1	0	1	18	18	9	20	26
3	4	4	4	4	1	22	20	16	14	32
5	5	3	3	0	1	16	14	9	14	27
3	5	3	3	0	2	20	16	13	15	29
4	4	3	7	4	1	23	19	18	15	40
4	3	3	7	4	1	24	17	16	12	38
4	5	3	5	2	1	18	18	13	17	32

4	3	3	4	4	1	24	16	14	14	31
4	3	3	6	4	1	22	20	17	12	37
5	2	4	7	4	2	22	18	19	20	33
3	3	3	3	1	1	18	16	14	16	28
5	3	3	7	3	2	26	16	14	16	31
5	4	3	5	4	1	22	17	15	18	34
4	3	3	4	3	1	19	12	15	18	29
2	4	3	4	1	1	19	13	14	14	26
4	5	3	3	4	1	23	18	19	16	36
4	2	4	4	4	1	22	17	18	15	30
4	3	4	1	4	1	20	16	17	14	36
5	3	4	5	4	1	21	17	15	13	36
4	4	2	3	1	1	21	16	14	19	29
2	3	3	3	1	1	20	16	13	16	29
6	2	2	2	1	1	12	17	8	16	23
5	4	3	7	4	1	16	15	14	15	36
3	2	4	3	4	1	18	15	15	14	29
5	3	3	4	1	1	16	16	12	12	25
5	3	4	4	1	1	17	17	15	18	28
2	3	3	3	2	1	19	19	16	18	29
2	5	3	3	2	1	18	14	14	14	39
5	4	4	5	0	1	23	17	15	20	37
5	1	4	4	4	2	19	18	17	17	28
4	5	3	3	3	2	26	16	17	15	37
3	3	3	4	1	1	16	17	10	18	28
4	5	3	4	0	1	22	17	9	14	28
6	4	3	7	4	2	25	16	9	17	45
3	4	3	2	2	1	23	20	13	10	24
4	4	3	6	4	1	27	14	18	19	34
3	5	3	3	1	1	23	14	14	15	28
5	5	3	4	3	1	16	19	12	17	30
4	5	3	5	3	1	19	17	14	16	32
4	4	3	5	3	2	22	19	15	13	37
2	3	3	2	1	1	14	17	10	12	25
3	5	3	3	0	1	20	10	9	17	28
4	5	2	3	0	1	13	12	8	17	29
2	3	2	2	3	1	16	14	15	15	31
3	3	4	1	0	1	10	9	6	16	26
2	3	3	3	3	1	18	14	11	15	28
3	4	1	1	0	2	14	16	11	14	23
4	5	3	5	3	1	19	17	15	14	34
6	1	4	2	0	2	16	15	15	15	23
4	4	3	7	4	2	22	15	15	15	34
3	4	3	1	4	1	24	17	18	16	28
4	4	3	7	3	1	21	18	11	15	36
4	3	3	5	4	1	15	15	17	13	33
5	4	3	4	0	2	21	16	14	17	28
5	4	3	7	4	1	18	17	13	13	35
3	4	3	1	4	1	12	11	14	14	29

6	3	3	6	4	1	19	16	19	20	31
5	3	3	7	4	1	17	18	13	9	42
4	3	3	6	2	1	22	20	12	16	34
4	3	2	6	3	1	19	14	12	16	30
5	4	3	7	4	1	21	20	18	17	45
2	3	3	2	1	2	21	16	16	17	25
3	3	3	7	4	1	16	18	18	17	33
4	3	3	7	4	1	22	12	16	16	38
2	3	2	2	3	2	15	17	13	12	26
1	1	2	2	2	2	18	19	16	17	26
2	1	3	3	1	1	12	17	6	13	24
4	3	3	6	1	1	18	18	13	18	24
2	3	3	5	3	1	18	18	16	16	36
2	3	3	3	1	1	12	5	6	15	25
3	3	3	5	1	1	17	10	10	10	32
2	3	3	2	1	2	13	13	15	16	36
6	2	4	4	4	2	23	14	14	18	35
4	4	3	4	3	1	23	16	11	16	22
2	2	1	1	0	2	18	17	13	17	16
5	1	4	2	0	1	17	16	14	18	15
1	2	2	2	1	1	11	11	8	13	21
3	3	3	4	4	1	21	19	16	12	36
4	3	4	3	0	1	26	15	12	18	25
5	4	4	3	4	1	23	18	19	18	23
5	3	4	3	0	2	21	19	13	15	31
4	3	4	4	4	1	20	17	17	15	29
6	4	4	2	4	1	19	13	12	20	28
5	4	4	2	0	1	18	17	12	16	20
5	2	4	2	0	1	16	17	13	18	21
4	3	4	1	0	1	17	16	11	14	19
3	2	4	6	4	2	28	19	19	18	45
3	4	4	2	0	1	18	13	8	16	26
3	3	4	3	0	2	15	18	14	19	26
6	1	4	2	0	1	19	13	11	18	21
5	3	4	2	4	2	21	15	18	20	23
2	2	3	3	1	2	19	19	16	20	31
6	1	4	3	0	2	16	17	11	14	18
3	4	4	3	4	1	23	19	19	18	30
6	4	4	7	4	1	14	14	11	14	40
4	3	4	7	0	2	22	20	15	15	32
5	3	3	3	0	1	15	18	7	13	18
5	4	2	4	0	2	20	17	7	19	23
4	2	3	7	3	1	24	13	14	17	32
5	3	3	6	4	1	24	19	19	19	24
2	3	4	2	0	1	21	13	13	14	29
4	3	4	2	0	1	13	18	15	13	17
5	1	3	6	4	2	17	18	17	17	36
4	3	4	2	0	1	12	17	13	19	17
4	2	3	3	1	1	15	17	11	13	25

4	1	4	2	0	1	21	17	7	19	18
5	1	4	2	1	2	20	19	16	20	19
3	3	4	7	0	1	25	15	9	14	24
5	3	4	3	0	1	22	16	13	12	25
5	2	1	1	0	1	18	18	15	17	19
5	5	4	7	4	1	21	16	11	16	34
5	3	4	4	0	1	22	19	14	20	21
5	2	4	4	4	2	23	19	16	18	38
6	4	4	5	4	1	23	19	19	16	29
4	2	4	4	4	2	16	18	17	18	38
5	3	2	3	1	1	16	18	15	16	17
2	4	3	7	1	2	22	20	16	13	41
5	3	4	2	4	1	12	15	16	17	22
4	3	4	4	4	1	19	15	17	16	32
5	3	3	4	2	1	18	19	9	15	22
6	3	3	4	1	1	21	18	11	19	22
4	3	4	2	0	1	22	15	12	17	20
4	4	4	6	4	1	24	17	19	12	30
3	3	3	5	3	1	17	14	15	18	26
4	3	4	4	0	1	15	16	14	16	33
5	3	4	2	0	1	22	15	8	16	17
4	3	4	1	0	2	22	16	14	19	20
4	2	3	4	4	2	21	20	16	17	30
5	4	3	4	4	2	19	19	11	13	35
5	4	4	4	0	1	17	20	10	17	23
5	4	4	3	4	1	21	17	12	15	27
4	4	4	3	0	1	22	17	12	17	23
3	2	4	3	4	2	13	17	16	10	25
3	3	3	3	3	1	17	20	14	16	30
4	4	3	5	4	1	16	18	18	18	28
3	4	4	1	4	1	24	17	19	14	26
5	1	3	3	1	1	16	14	9	14	20
3	3	3	4	3	1	18	17	17	15	33
5	2	3	3	3	2	27	18	18	12	25
3	3	3	5	3	2	14	18	16	18	35
4	4	3	5	4	2	25	19	18	19	29
3	3	3	4	4	2	20	18	16	18	30
4	3	4	7	4	1	17	9	17	7	26
3	4	3	4	1	1	15	13	10	16	25
4	5	4	4	3	1	25	18	15	14	25
4	4	4	7	4	1	20	18	17	17	35
5	3	3	5	3	1	19	14	10	14	25
2	3	3	3	2	1	20	12	13	17	22
5	2	4	3	4	1	12	12	12	16	24
4	3	4	3	1	1	13	12	5	18	31
5	4	4	3	4	1	17	18	15	14	33
5	4	2	2	3	1	24	15	13	15	21
5	4	2	2	0	2	16	19	10	8	24
2	3	4	2	0	1	14	14	5	15	20

3	3	3	6	3	1	19	14	15	17	23
4	4	3	4	0	2	21	16	11	15	24
5	4	3	6	4	1	26	14	14	18	28
4	1	4	2	3	2	18	15	13	13	23
5	4	3	5	4	2	26	14	19	17	39
2	4	2	2	1	1	17	14	12	14	27
2	4	3	2	4	1	19	20	16	13	28
2	4	3	2	3	1	16	16	12	18	24
2	3	3	2	1	1	16	15	11	18	30
3	3	3	4	4	1	16	17	14	17	28
5	3	3	6	3	1	22	15	13	16	38
2	4	2	2	1	1	19	16	13	20	26
4	3	3	7	1	1	21	16	13	13	36
3	2	2	2	3	1	15	17	13	13	21
5	4	3	5	4	1	19	20	17	16	26
5	4	3	6	4	1	24	18	16	19	35
4	2	3	5	4	1	14	18	17	16	32
3	4	3	3	3	2	20	16	15	14	32
3	4	2	2	0	1	20	13	9	16	16
4	4	3	5	0	2	18	16	11	15	30
3	4	3	2	3	2	20	20	14	14	22
4	3	3	3	4	1	23	18	17	16	41
2	3	2	2	2	1	20	19	13	18	19
3	3	4	3	4	1	22	20	17	13	37
4	4	4	1	0	1	23	16	13	15	25
4	2	3	5	2	1	18	16	13	15	29
5	1	3	4	3	2	16	15	12	16	33
4	3	3	7	4	1	16	17	17	18	33
3	3	3	6	1	1	15	19	13	15	38
2	3	3	2	0	1	14	16	10	10	17
3	2	2	3	1	1	14	19	11	20	18
5	2	4	1	3	2	15	20	18	18	26
4	4	3	5	4	1	17	18	14	17	31
3	4	3	3	1	1	15	16	8	17	31
4	5	3	3	0	1	24	14	10	12	21
3	5	3	4	1	1	16	17	6	8	26
4	4	4	6	4	1	23	20	18	19	37
6	5	4	2	0	2	18	13	10	18	26
2	2	2	1	1	2	18	8	10	15	22
3	5	2	2	0	1	13	16	9	9	38
2	3	3	2	2	1	18	15	12	19	24
2	3	2	2	1	2	15	17	12	15	26
3	5	3	4	1	1	17	14	7	14	27
3	4	3	5	2	1	21	15	15	19	22
3	4	3	6	2	2	21	18	14	16	31
4	5	3	3	2	1	22	19	15	17	22
2	4	2	2	2	1	16	16	10	14	19
5	2	3	5	4	1	18	20	16	18	35
5	4	3	4	2	1	18	16	11	17	26

4	4	3	7	3	1	26	18	12	15	37
2	1	2	2	2	1	17	15	14	17	15
2	3	1	1	0	1	11	16	5	14	20
4	4	3	4	2	1	22	16	14	17	25
5	2	4	2	0	2	21	20	13	17	24
5	3	4	3	0	2	16	18	12	17	27
3	4	3	2	1	1	20	16	13	13	18
4	3	4	2	3	1	16	11	13	18	21
3	4	1	1	0	1	15	16	12	15	20
5	3	4	5	4	1	20	19	16	19	24
5	3	3	7	4	1	26	18	15	20	39
4	3	1	1	0	2	19	17	14	16	15
3	4	3	2	0	1	20	14	8	15	21
3	4	1	1	0	1	14	18	11	18	16
4	4	4	3	4	2	23	19	18	16	26
2	2	3	3	2	1	19	18	15	16	19
5	3	3	6	3	2	14	16	16	15	33
5	4	4	5	0	1	23	18	15	19	29
4	3	3	4	2	1	19	18	12	15	22
2	4	2	3	3	1	14	17	17	13	20
4	5	3	6	3	1	25	14	13	17	35
3	5	3	3	1	1	21	11	7	17	22
3	4	2	2	0	1	26	15	13	15	18
3	4	3	3	3	2	20	17	16	17	22
4	4	1	1	0	1	14	13	8	18	19
5	2	4	4	0	1	20	19	15	16	18
3	5	3	3	3	1	18	15	15	19	31
3	4	2	2	1	1	14	10	9	17	22
5	5	3	6	4	1	20	12	11	17	35
2	4	3	2	1	1	18	14	12	16	23
3	5	3	5	0	2	20	16	14	15	26
2	4	3	2	0	1	14	14	10	19	23
4	2	4	2	4	1	14	17	13	13	26
2	1	3	2	0	1	13	16	10	14	24
2	3	3	3	3	1	22	19	17	16	36
4	3	4	2	4	1	19	16	14	13	22
4	3	3	4	1	1	21	15	12	16	23
4	3	3	4	4	1	20	12	12	15	37
4	5	1	1	0	1	17	12	10	12	17
3	2	3	3	1	2	20	18	13	16	26
4	4	3	5	3	1	21	16	11	18	24
5	3	4	4	3	1	15	15	13	16	26
4	1	3	3	2	1	17	14	12	17	22
2	4	2	2	1	2	17	16	12	13	22
2	4	2	3	1	1	21	18	14	18	19
3	3	3	2	3	1	18	17	13	17	32
3	3	1	1	0	1	12	16	11	7	16
3	5	3	4	2	1	23	16	13	19	30
4	4	3	4	1	1	17	15	12	14	21

2	3	3	2	3	1	18	14	14	15	28
2	2	3	3	1	2	19	16	13	14	23
2	3	3	2	0	1	20	15	10	18	16
2	4	3	3	0	2	17	14	12	19	25
2	4	3	2	3	1	22	19	12	14	24
3	5	1	1	0	1	19	16	11	15	18
3	4	4	5	3	1	20	15	14	18	25
4	5	3	4	0	1	15	13	5	16	25
3	1	4	3	4	2	20	17	16	17	20
1	1	2	1	1	1	10	14	10	12	26
2	4	3	4	1	1	21	10	5	17	23
4	4	3	3	3	1	22	14	10	16	27
5	3	3	3	1	1	17	19	13	16	30
2	3	3	3	1	1	20	15	12	18	20
6	3	3	3	1	2	19	15	12	17	21
3	4	2	2	0	1	14	13	11	16	23
2	4	3	2	1	1	20	17	14	15	25
3	4	3	2	0	2	19	15	12	18	19
4	2	3	3	1	1	13	17	7	11	20
2	4	1	1	0	1	13	13	10	14	19
6	5	3	4	2	1	20	20	13	19	26
4	1	3	4	2	1	12	14	12	13	29
5	4	2	2	0	1	15	11	11	18	16
3	3	3	3	1	1	15	15	10	12	18
3	3	3	4	1	1	19	16	14	16	31
3	3	3	3	1	1	19	12	8	17	18
3	3	1	1	0	1	11	9	5	13	16
2	4	2	2	1	1	15	17	10	15	19
3	4	2	2	0	1	13	13	10	15	20
2	4	3	2	1	1	22	18	15	18	20
2	3	2	2	1	1	15	12	10	11	19
3	4	3	4	1	1	18	14	11	14	23
2	3	3	2	3	1	16	13	6	18	23
4	5	3	7	3	1	18	14	9	18	26
4	4	3	6	0	2	18	17	14	13	21
2	4	2	2	1	1	18	16	9	14	21
3	3	3	4	1	1	19	13	10	10	24
2	3	3	3	2	1	23	17	14	20	27
2	1	3	3	3	1	17	15	12	14	31
3	4	3	2	0	1	14	18	7	17	21
4	3	3	3	1	1	18	18	8	19	33
3	3	3	4	2	1	21	17	17	17	20
3	3	3	3	3	1	16	13	11	15	36
2	3	3	2	1	2	18	14	13	19	17
4	5	3	5	0	1	19	13	11	16	22
5	5	3	5	3	1	17	9	7	15	24
4	3	3	4	3	1	21	16	13	17	22
3	3	3	6	4	1	22	19	19	19	37
4	5	3	4	1	1	15	14	10	11	26

3	3	3	3	2	2	14	13	10	15	19
2	3	1	1	0	1	16	18	11	14	22
5	1	1	1	0	1	16	9	10	14	11
6	5	3	5	0	1	22	15	10	18	23
4	3	3	3	4	1	21	20	15	20	33
5	5	3	4	0	1	16	18	12	11	28
4	5	2	2	4	2	18	12	16	20	28
2	3	2	2	2	1	17	18	15	17	28
4	4	4	2	0	1	15	11	7	11	26
5	1	3	3	2	1	17	15	14	15	26
4	4	4	6	4	1	22	20	19	18	30
5	2	3	5	1	1	16	14	5	15	22
5	4	4	4	0	1	20	18	14	15	28
4	5	3	5	3	1	21	17	16	20	26
4	3	3	5	3	1	21	16	16	16	30
3	4	3	5	3	1	23	14	15	15	27
2	3	4	3	0	1	17	18	11	16	32
5	2	3	2	1	1	11	14	6	16	17
3	4	3	4	1	1	16	12	5	14	33
3	4	3	6	3	1	28	20	17	18	37
3	6	3	4	3	2	21	20	18	11	32
3	5	3	4	2	2	18	18	14	19	24
3	3	2	2	1	1	16	18	14	16	20
3	3	3	4	4	1	19	16	15	17	26
3	3	3	4	3	2	24	13	14	15	23
3	3	3	3	4	1	18	14	17	11	30
3	4	3	7	4	2	21	14	15	19	30
4	4	3	5	3	1	19	17	14	16	28
4	2	3	7	3	1	14	15	15	16	37
4	3	4	2	2	1	20	17	14	14	30
3	3	3	5	2	1	17	17	10	10	37
4	4	3	5	3	2	24	17	15	14	25
3	4	3	4	3	2	24	19	15	20	25
5	3	4	3	1	2	21	19	16	15	28
1	1	2	2	1	1	12	15	11	13	18
2	2	3	3	1	1	16	16	11	15	22
3	4	3	4	0	1	16	18	12	14	28
3	2	3	3	1	1	15	16	7	13	26
2	3	3	3	1	1	21	16	13	17	23
2	2	3	2	2	1	16	18	12	12	28
3	5	3	4	3	1	21	10	13	15	25
2	2	4	2	0	1	19	16	12	15	24
3	3	3	4	3	1	21	17	13	18	29
3	2	1	1	0	2	16	19	12	12	18
3	3	3	4	1	1	18	17	11	16	27
4	3	3	7	3	2	21	19	16	17	26
3	5	3	5	1	1	17	13	11	16	23
4	2	2	2	1	1	10	11	7	13	26
3	5	2	2	0	1	20	15	11	18	17

4	4	4	3	4	1	18	15	14	15	29
3	5	4	4	0	1	15	15	6	14	34
2	3	2	2	2	1	19	20	14	14	17
3	5	3	2	1	1	22	14	12	11	24
3	5	4	2	2	1	19	16	13	17	24
2	6	2	2	1	2	22	12	11	17	20
2	3	3	4	1	1	13	17	12	15	32
5	5	4	2	4	1	21	17	17	15	31
5	3	4	7	4	1	18	15	19	16	42

RSES Sum	BSRI-M MEAN	BSRI-F MEAN	BSRI-N MEAN	SNI SIZE	SNI DIV	DOM ROLE_SEX	ENJOYS SEX	INTIATES SEX	LIFE SAT	SEX FREQ	SEX PREF	SEX EXP
42	4	4.2	4	37	6	3	5	3	4	3	1	4
45	4	4.6	4.6	29	9	2	3	2	3	1	1	2
35	4	4.2	4.2	28	7	3	4	2	3	1	1	2
49	3.8	4.4	4	24	8	2	5	3	3	1	1	2
43	3.4	3.2	3.8	31	7	3	4	3	3	1	2	2
41	3.8	4.6	4.8	26	6	3	5	3	3	2	2	2
34	2.6	4.6	4.8	48	8	2	3	2	3	2	1	2
44	3	4.6	4.2	40	12	2	3	2	3	1	1	2
35	4.2	4.4	4	11	5	2	4	3	3	0	1	2
42	3.4	3.2	4.2	10	7	1	2	1	2	0	1	0
40	3.6	4.4	4.2	15	5	1	4	2	2	0	1	2
39	3.8	4.6	4.8	39	8	2	3	2	3	1	1	2
37	3.8	4	4.2	18	5	2	3	2	2	2	1	2
46	3.4	4.2	3.8	36	9	1	3	1	3	0	1	0
47	4.2	4.6	4.4	21	7	2	4	3	2	0	1	2
43	3.6	3.8	4.4	29	8	3	5	3	3	1	1	0
40	4.6	4.2	4.6	18	6	3	5	3	3	1	1	4
44	3.8	4.4	4.4	22	10	3	5	4	3	1	1	0
37	4.6	5	5	27	12	3	5	3	2	4	1	2
45	3.4	4.4	3.8	14	7				3			
42	4.4	3	3.8	36	8	4	5	5	2	2	1	2
30	3.2	5	4.6	15	8	2	4	4	2	2	1	2
44	4.8	2.4	4.4	18	5	3	4	4	3	0	1	3
49	4.8	5	4.8	15	6	1	5	1	3	1	1	1
45	3.4	4.4	4.4	27	9	3	4	2	3	1	1	0
44	3.8	3.8	4	38	9	2	4	2	3	1	1	2
32	3.6	4.2	3.4	12	5	2	5	2	2	1	1	3
40	3.6	4.8	4.6	41	10	2	5	3	3	2	1	1
34	3.6	4.4	4.6	36	11	1	2	2	2	1	1	1
39	3.8	4.4	4.8	28	9	2	4	2	4	2	1	0
40	4	4.6	5	45	8	2	5	3	2	0	1	4
45	3.6	3.4	4.8	27	10	2	3	1	1	1	1	2
34	3.8	3	3	20	5	2	3	3	2	1	1	2
48	4.2	3.6	4	22	7	1	4	2	4	2	1	2
48	4	3.8	4.4	27	7	2	4	3	3	1	1	2
44	3.2	4	4	30	7	3	5	3	3	0	1	2
43	3	4.2	4.2	29	6	1	2	1	3	0	3	1
32	4.2	3.2	3.6	30	8	1	3	2	3	0	1	0
43	4	5	4.8	38	9	2	5	2	3	2	1	0
35	3.4	4.2	4	25	7	3	4	2	2	0	1	1
39	4.2	4.8	4.4	36	10	2	2	2	3	0	1	0
42	3.6	4	4.2	19	8	2	4	3	3	1	1	2
26	3.4	4.2	3.8	20	6	3	5	3	1	4	1	1
50	4.4	4	4.4	41	9	2	3	2	3	0	1	2
44	3.8	3.6	3.8	17	5	2	3	2	3	1	1	2
44	3.8	4	4.4	22	7	3	5	3	3	1	1	1

33	3.2	3.6	4	12	4	2	4	2	2	0	1	3
41	4.2	3.4	3.2	17	7	2	4	1	3	1	1	2
48	4.8	4.4	4.4	32	9	2	5	2	4	1	1	0
31	3.6	4.2	4.2	32	8	1	5	2	1	0	1	2
48	4.4	4.4	4.8	18	5	3	5	3	3	1	1	3
48	3.8	4.2	4.8	30	8	3	3	3	3	1	1	0
26	3	4	3.8	31	6	2	5	3	1	0	2	3
29	4	3.8	4.2	21	6	1	2	2	2	1	1	3
47	3.8	3.8	4	36	10	2	5	2	3	1	1	2
43	4	4.4	4.6	33	8	2	5	3	3	0	1	2
43	3.6	4	4.2	22	7	3	4	3	4	4	1	1
35	3.8	4	4	27	9	1	4	3	1	1	1	3
42	3	4.8	4.6	22	7	1	5	3	3	1	1	0
40	3.4	4.4	4.2	28	7	2	4	3	3	1	1	2
39	3	3.8	3.8	27	9	2	5	1	3	0	1	3
41	3.8	4.6	4.4	15	4	2	4	1	3	1	1	0
44	4.2	4.4	3.8	11	4	2	4	2	3	0	1	3
39	3	3.4	3.8	17	7	3	5	3	2	1	1	0
42	3.6	4	4.4	51	11	3	5	4	4	1	1	2
34	4.6	4.6	4.6	24	8	2	5	3	2	4	3	3
29	2.8	4.2	3.8	26	7	1	4	1	2	4	1	0
43	3.2	4	4.4	20	6	2	3	2	4	1	1	2
43	3.8	3.6	3.8	18	7	2	5	2	3	0	1	4
46	4.2	3.6	4.4	19	8	3	4	3	3	1	1	2
29	3	4	4	21	7	4	4	4	2	2	1	2
40	3.6	4.2	4.2	17	8	2	1	1	3	1	1	2
50	4	4	4	18	4	3	3	2	3	0	1	3
39	4.6	4	4.2	11	5	4	5	3	2	0	2	2
44	3.6	4.6	4.2	17	7	2	3	3	2	1	1	3
39	4	3.2	4	17	4	2	5	2	3	1	1	2
39	3.6	3.8	4	34	6	2	4	3	3	0	2	2
48	2.8	4	3.8	23	9	2	3	2	2	1	1	2
44	4	3.8	4.4	17	7	3	5	3	3	1	2	4
33	3.6	3.6	3.6	17	7	1	4	3	2	0	3	2
43	3	4.8	4.6	22	7	2	4	2	3	0	1	0
32	3	4.2	4	9	6	3	5	3	3	2	1	2
33	3.6	4.6	4.2	18	6	1	3	1	2	0	1	0
24	2.6	3.8	3.6	8	4	1	0	1	1	0	1	0
43	3.2	3.8	4.4	21	4	2	3	2	2	1	1	0
31	3.6	4.8	4.6	23	8	2	5	4	3	1	3	2
40	3.4	3.6	4	20	6	2	4	2	3	1	1	2
38	3.6	5	5	10	3	2	4	2	3	1	2	3
43	4	3.8	3	15	4	2	5	3	3	0	1	1
38	4	4.2	4.2	28	9	2	4	2	3	0	1	2
45	4	5	4.8	23	8	2	4	3	4	4	1	0
37	3.4	4.2	4	19	8	2	4	2	2	1	1	2
43	3.4	5	4.8	26	8	2	3	2	3	2	1	3
46	3.6	4.2	3.8	20	7	3	3	3	2	1	1	3
40	3.4	4.2	4.2	21	6	3	5	3	2	4	1	4

39	3.6	3.8	4.4	15	4	2	3	2	2	0	1	4
37	4	2.6	4	14	4	2	4	2	1	0	1	3
45	3.8	4.6	4.8	25	8	3	5	2	4	0	2	1
40	3	4.2	4.2	26	7	2	5	3	3	0	1	0
46	4.2	3.8	4.4	35	7	3	5	3	4	2	1	2
41	4.6	4.2	4.4	20	5	3	4	4	3	4	1	3
49	3.6	4.6	4.2	17	7	2	5	3	4	2	1	2
45	3.4	2.8	4.2	15	5	3	4	2	3	1	2	2
38	4.4	3.2	4.4	16	5	2	4	3	3	0	1	0
40	4	4.8	4.8	28	6	1	5	1	4	0	1	0
24	3	4.4	3.8	19	9	3	5	2	2	1	1	0
42	3.8	3.8	4.2	23	10	3	5	4	2	1	1	2
39	4	4.6	4.4	16	7	3	5	3	3	3	1	2
36	2.8	4.6	4	15	7	3	3	3	2	1	1	2
28	1	1	1	3	2	1	1	1	0	1	2	0
21	4.4	4.2	4	38	7	5	5	4	2	2	1	2
40	3.4	3.4	4	16	5	4	4	3	3	1	1	1
45	3	5	5	17	7	2	5	2	2	0	1	0
35	3.4	4.8	4.6	27	5	3	5	3	3	2	1	0
24	3.4	4	4	27	7	2	4	2	2	0	1	3
22	3.2	3.2	3.8	17	5	3	3	3	1	1	1	2
44	4.2	4.8	4.8	29	7	3	5	3	3	3	1	4
40	3.6	4.8	4.4	35	9	3	5	2	3		1	2
41	3.4	4.4	4.4	15	6	3	5	3	3	4	1	1
40	4	3.8	4.4	36	9	3	4	3	3	1	1	1
43	3.8	4.6	4.4	28	8	3	5	3	2	1	1	2
41	2.6	3.8	3.6	25	7	3	4	3	3	1	1	1
35	3.4	4.2	4	9	2	3	5	3	2	0	1	2
35	3.2	4.6	4.4	17	7	3	1	3	3	0	2	2
39	3.4	4.8	5	22	8	2	5	3	4	2	2	2
44	3.8	4.8	4.4	48	10	2	5	4	4	4	1	3
36	3.2	3.4	3.8	31	9	4	3	4	3	1	1	0
35	3.8	4	3.8	21	8	2	5	3	3	2	1	2
32	3.4	3.8	4	19	7	2	4	2	2		1	2
41	3.2	4.6	4.6	41	8				2			
34	4.6	4.6	4.4	23	6	2	5	5	3	4	1	3
34	4	4.4	4.4	19	5	3	5	3	3	1	2	3
43	3.6	4.4	4.2	15	4				3			
38	3	3.8	4	16	5	4	4	4	2	1	1	3
45	3.8	3.8	4	15	4	2	5	3	4	4	1	2
41	3.8	4.6	4.4	20	5	3	5	3	3	1	1	2
39	4	4	4	17	9	1	3	2	2	1	1	2
45	2.8	4	4	26	8	2	4	3	2	1	1	2
43	4.4	4	4	40	8	2	3	2	3	1	1	0
22	2.2	3.8	3.6	11	3	5	2	1	0	0	1	0
38	3.6	5	5	20	8	3	5	3	4	1	1	1
40	3.8	3.8	4.8	15	6	4	5	2	2	1	1	2
39	3.2	4.4	4.6	22	7	2	5	2	3	2	1	2
32	3.4	4.2	4.2	7	4	2	1		3	0	2	2

37	3	4	4.4	28	7	2	3		3	1	1	2
39	4	4.6	4.6	17	6	2	5	2	4	0	2	4
44	4.2	4.4	4.8	22	8	3	5	3	3			0
40	3.6	4.4	4.6	15	6	5	5	3	4	1	2	3
29	3.8	4	4.2	31	5	3	5	3	2	3	1	2
51	3	4	4.4	15	6	3	5	3	3	1		2
41	3.4	5	4.6	15	5	2	5	4	3	0	1	3
41	4.2	4.4	4.2	24	7	3	4	2	3	1	1	2
48	4.2	4.8	4.6	38	7	2	5	2	3	0	1	3
44	4.4	4.4	4	12	4	2	4	2	3	1	1	4
42	4	5	4.6	38	7				2			
47	4.4	4	4.8	23	4	3	5	3	3	1	2	3
40	3.4	4	4	28	7	4	5	4	2	1	1	1
39	3.8	3.4	3.8	39	10	3	5	3	3	2	1	2
42	3	4.2	4	24	7	1	3	2	3	2	1	2
43	3.6	4	4	51	8				3			
39	3.6	4.2	4.6	24	7	3	5	2	4	1	1	0
49	4.2	4.6	4.4	19	11			3	3		1	
44	3.8	4.8	4.6	16	6	2	5	3	4	4	1	0
37	3	4.4	4.4	20	7	2	5	4	2	0	1	1
40	3.4	4.8	4.8	18	4	2	4	3	4	2	1	2
36	4.4	4.2	4.6	9	4				3			
44	4	3.6	4.6	40	7				2			
47	4.2	4.8	4.8	10	3	1	5	1	4	0	1	4
43	4	4.4	4.8	23	7	2	4	2	4	2	1	2
43	3	4	3.8	17	4				2	0	1	
31	3.6	4	4	22	9	2	4	3	3	1	1	0
43	4.2	4	4.2	15	6	1	5	1	3	0	1	2
42	3.8	4	4.6	18	7				3			
46	3.6	4.4	4.8	37	10	1	4	2	3	2	1	0
39	4.4	3.4	4	16	7	2	5	3	2	1	1	1
31	3.2	4.4	4.2	25	8	2	5	3	1	1	1	2
45	4	3.4	4.6	11	6	2	5	2	3	2	1	1
39	4.6	4.6	4.6	19	9	2	5	4	1	1	1	3
37	4.6	4.2	4.6	27	8	3	5	4	3	1	1	2
46	4.2	4.6	4.4	19	6	4	5	4	2	0	2	4
38	4.6	3.8	4.2	26	7	4	5	3	3	3	1	4
44	3.2	4.4	3.6	18	8	1	4	2	3	1	1	2
40	2	4.6	4.2	12	6	3	4	3	3	1	1	1
34	3.2	3.6	3.8	11	3	4	3	4	1	1	3	3
45	4	4.2	4	22	4				2			
35	3.6	3.4	3.4	18	4	2	4	2	2	0	1	2
36	3.2	3.8	3.8	24	5				2			
41	3.2	4.2	4.2	16	4	2	5	3	3		1	2
28	3.4	3.2	4	7	4				1			
39	4	4.2	4.8	9	5	2	3	4	2	1	1	1
33	3.4	4	3.8	29	7	2	3	2	2	1	1	2
29	3.2	3.8	3.8	27	9	3	5	1	2	0	1	3
36	3.6	3.8	3.6	13	5	3	3	2	3	2	1	1

40	3.6	4	3.8	18	6	2	4	2	2	1	2	1
37	3.6	4	4.2	23	6	3	4	3	2	2	1	3
42	3.4	3.6	3.4	19	6	2	4	2	2	1	2	2
32	3	4.2	3.8	27	9	3	5	3	3	1	1	1
47	3.4	4	4.4	21	8	3	4	4	4	0	1	1
24	3.6	3.6	3.6	14	4	2	4	2	2	0	1	2
36	3.8	5	4	21	7	2	2	4	2	1	1	0
33	3.4	4.2	4.2	14	7	3	4	3	3	1	1	0
29	3	4.8	4.2	27	5	2	5	3	2	2	1	2
42	3.8	4	4	28	8	1	4	2	3	2	1	2
41	3.6	4.2	4.6	12	5	3	5	3	3	1	1	2
32	3	3.4	4.4	36	7	3	5	3	3	4	1	2
41	3.8	3.2	3.6	23	8				3			
30	3.4	5	4.2	16	4	1	1	1	2	0	1	0
43	4	3.6	4.6	16	6	3	5	4	3	1	1	2
37	3.8	3.8	4.4	20	10	2	2	2	2	1	1	2
37	3.6	4.2	4.4	25	6	2	3	2	3	0	1	1
40	4.4	4.4	4.2	13	7	4	4	4	2	1	1	0
29	2.6	4.2	4.4	16	8	2	3	3	2	0	1	2
32	4	4.2	4	18	6	3	5	3	2	3	1	2
38	3.8	4.6	4.8	20	3	4	5	4	3	4	1	1
35	4.2	3.8	4.6	17	7	3	5	3	1	2	1	3
40	4.6	4.2	4.4	35	10	3	5	3	3	2	1	3
42	4.2	4.2	4.2	27	9	2	4	2	3	1	1	1
34	4	3.6	3.6	44	10	3	5	3	3	1	1	3
39	3.2	4	3.8	38	7	2	4	3	3	1	1	2
39	3.4	3.6	4.2	17	6	1	3	2	2	1	1	2
31	3.8	3.8	4.2	18	5	3	5	3	1	3	1	3
32	3.8	3.4	3.4	5	2	1	3	2	2	3	1	0
34	3.2	3.4	4.6	21	5	2	5	3	3	1	3	2
40	3.6	5	4.8	18	5	3	5	3	4	1	3	2
41	4.6	4	4.4	14	5	3	5	2	4	4	1	1
42	3.4	4	4.4	10	4	1	4	2	2	1	1	3
32	3.6	4	3.8	18	6	2	4	2	2	2	1	2
29	3.4	3.6	4	6	3	2	4	2	2	0	1	2
36	2.8	4.8	4.4	15	6	2	5	3	3	3	2	2
48	4	4	4	21	8	2	5	4	3	1	1	1
37	4.2	4.2	4.2	14	5	2	5	4	3	1	1	1
19	3	4.6	4	8	4	2	5	2	0	0	1	0
42	3.4	4.2	4.4	14	7	2	3	3	3	1	1	2
35	3.8	3.8	4	12	5	2	4	2	2	1	3	2
35	4.2	3.4	4.2	17	5	3	5	3	4	1	1	2
29	2.8	4.2	3.2	19	7	1	2	1	2	0	3	2
37	3.6	4.6	4	15	6	3	2	3	2	1	3	0
44	3.8	4.4	4.4	21	4	3	5	4	3	1	1	2
45	3.8	4.4	4.4	30	9	3	5	3	3	2	1	2
39	3.4	4.4	4.4	16	5	2	5	3	3	1	1	0
41	4	4.6	4.8	30	5	2	5	3	3	1	1	3
45	3.4	4.6	4.2	18	6	1	5	3	3	2	1	1

44	3.6	3.8	4.4	23	7	3	5	3	3	3	1	1
32	3.8	4.4	3.8	31	9	2	5	3	2	3	3	1
22	2.6	3.8	3.4	14	5	1	5	3	2	3	1	2
45	3.4	3.8	4.6	23	10	2	3	2	3	1	2	2
37	3.8	3.6	4.2	21	6	2	3	2	4	2	1	1
41	4	3.8	3	7	2	3	5	3	2	4	2	4
40	4.2	3.6	4.2	12	4	2	2	2	3	0	1	3
34	3	3.8	3.8	24	10	1	3	2	2	1	1	2
31	3.6	3.4	3	18	6	3	4	1	2	0	1	2
47	3.6	5	4.8	18	6	3	5	3	2	2	1	1
40	3	4.4	4.6	33	7	3	5	3	3	2	1	1
39	3.4	4.4	4.4	41	9	3	5	3	3	2	1	1
33	3.6	4.4	4.4	30	7	3	5	4	3	1	1	2
31	4.2	4.8	4	25	5	3	4	2	4	1	1	2
46	4.2	3.8	4.2	26	7	3	4	3	3	1	1	2
42	3.8	4.6	4.2	21	6	3	5	3	3	3	1	2
47	4	3.8	3.8	44	10	3	5	3	4	1	1	2
43	3.6	4.4	4.4	38	6	3	5	2	3	2	1	2
39	3.8	3.8	4	32	9	3	5	3	3	4	2	2
43	3.8	4.8	4.8	30	6	3	5	3	3	4	1	0
40	3.6	3.8	4.6	27	6	2	4	2	2	1	1	1
36	3.2	4	4.2	19	7	2	3	2	3	1	2	2
27	3.4	3.8	3.6	30	8	1	5	3	2	1	1	1
36	4.6	3.8	4.2	18	5	3	3	2	2	0	1	2
21	3.6	4	4.2	11	4	2	4	1	1	2	1	2
41	3.6	4	4.4	9	3	2	5	4	3	0	1	2
35	4	4.6	4.4	33	11	3	5	3	3	1	3	2
28	3.2	4	4.2	15	6	2	4	3	2	1	1	2
48	3.2	4.4	4.6	33	8	2	5	2	3	3	1	1
40	3.6	4.4	4	17	8	2	5	4	3	4	1	2
33	4	3.8	3.6	27	8	3	3	3	3	1	1	2
35	3.6	3.6	4.2	23	8	1	3	2	2	2	1	2
42	3.6	4.8	4.8	24	5	1	2	2	1	0	1	2
33	3.4	4	4.2	38	9	3	4	3	2	1	1	2
34	4.4	3.6	3.4	13	6	1	5	2	1	1	1	3
38	3	4.4	4.8	22	7	2	5	3	3	1	1	2
41	3.4	4.4	4	15	4	4	4	2	2	0	1	2
32	3.2	3.4	3.4	31	8	2	4	1	2	1	1	4
29	3.2	3.6	3.6	13	2	2	4	2	2	0	1	1
37	3.8	4	4.4	20	5	1	5	3	2	0	1	2
43	3.6	4.6	4.6	22	10	1	4	2	3	1	1	0
30	4	4.2	3.6	26	6	2	4	4	1	0	1	3
28	3.4	3.6	3.8	19	6	1	1	1	1	0	1	2
36	4.2	4.4	4.2	16	5	2	4	2	2	2	1	0
42	3.8	3.6	4	28	5	2	5	4	3	2	1	3
40	3.4	4.2	4.4	11	4	2	5	3	2	2	1	1
37	4	3.8	3.6	10	4	1	4	2	2	0	1	0
42	3.2	4.2	4.6	13	6	2	5	1	2	1	1	1
40	3.2	4	4.2	27	8	2	4	2	2	1	1	0

37	3	3.8	4	19	5	2	5	2	3	3	1	1
37	3	4.4	4.4	25	7	2	3	3	3	2	1	2
31	4	4.4	4.8	17	4	3	5	4	2	2	3	2
32	3.4	3.8	4.2	16	7	2	3	2	2	1	1	0
32	3.8	4.4	3.8	30	6	3	4	2	4	1	3	1
36	3.6	4	3.8	25	9	2	5	2	3	1	2	2
47	3.6	4.8	4.2	35	9	4	5	4	4	1	2	2
32	2.8	3.4	3.6	21	6	2	3	2	3	0	2	2
29	3.6	4.2	4	12	8	2	5	4	2	1	3	3
24	2.8	4	3.8	17	5	1	2	1	2	1	1	0
29	2.4	3.4	3.8	13	6	2	4	2	2	2	1	2
41	3	3.6	4	16	7	2	4	3	2	2	1	2
37	3.6	4	3.8	16	6	1	4	2	2	2	1	3
35	3.4	4.4	4.6	20	5	1	4	3	3	1	1	1
39	3.6	4.4	4.4	40	9	1	1	1	3	0	1	2
31	3.2	4.2	4.2	16	6	1	4	2	1	0	1	0
35	3.6	4.8	4.8	32	8	2	5	3	3	3	1	0
31	3.8	4.2	4	10	4	3	4	2	2	2	1	2
28	3.4	3.2	3.8	7	4	2	4	2	2	0	1	1
32	3.4	3.8	4.2	23	5	2	4	2	3	0	1	0
46	3.6	4.2	4.6	53	11	2	4	3	4	1	1	2
38	4.2	5	4.8	31	9	3	4	3	4	1	1	3
41	3.4	5	5	31	7	2	5	3	3	2	1	1
35	3.6	3.8	3.8	27	6	2	3	2	2	2	1	2
34	3.6	4.8	4.2	18	4	3	5	3	3	4	1	4
42	2.2	4	4.2	31	6	2	3	2	4	2	1	0
34	2.8	4.2	4.8	7	4	1	2	2	3	1	1	0
31	3.4	3.4	4.6	21	5	3	5	3	1	0	3	3
29	2.6	4.8	4.6	15	6	4	4	4	3	0	1	1
38	4.4	4	3.8	19	6	3	5	4	3	3	1	0
33	3.4	3.6	4	16	5	2	4	2	2	2	1	1
34	2.8	4	4.2	26	7	2	5	3	2	2	1	3
39	2.6	4	4	27	7	3	5	4	3	3	1	0
47	3.2	4	4.2	21	8	2	5	4	2	1	1	2
43	4	3.6	4	25	10	1	2	1	3	0	3	2
38	3.6	3.8	4.2	16	5	4	5	4	3	3	2	2
41	3.4	4.2	4.4	26	8	2	3	1	3	1	1	2
39	4	4.4	4.6	19	7	3	5	4	2	4	1	2
39	3.4	4	4.2	18	6	2	4	2	2	2	1	2
32	3.6	3.8	3.6	26	5	3	3	3	2	2	1	1
42	3	4.8	4.2	31	8	2	5	2	4	2	1	0
39	4.6	4.4	4.2	13	4	3	5	3	3	4	1	2
21	3.8	3	3.8	16	6	2	1	1	1	0	1	2
40	4.2	4.6	5	29	6	2	4	3	3	1	1	0
33	3.4	4	3.6	13	4	2	5	2	2	1	1	3
45	2.8	3.6	4.4	14	5	1	3	2	2	1	1	2
41	3.4	5	5	21	8	1	5	1	4	1	1	4
47	4.8	4.4	5	21	10	3	5	3	2	2	1	2
39	3	3	3.2	19	9	2	3	2	3	1	1	2

41	3.2	3.8	3.8	12	5	3	5	3	3	1	1	1
30	4.6	4.4	4.8	61	10	1	0	1	4	0	1	0
30	2.8	4.6	3.8	16	7	3	4	2	4	2	1	2
45	3.8	4.4	4.4	24	6	2	5	2	3	0	2	2
45	4.8	4.8	4.6	38	10	2	5	2	3	2	1	2
44	3.8	4.6	4.2	23	8	3	5	3	4	2	1	2
33	3.8	4.4	4.6	27	10	3	3	2	2	1	1	2
34	4.2	4.4	4.4	23	10	4	5	4	2	0	1	1
19	2.2	4	3.8	24	6	2	3	2	0	0	1	2
38	3.6	4.2	3.8	19	5	2	5	2	3	0	1	0
44	4	4.4	5	18	4	2	4	3	2	1	1	3
34	2.6	4.4	4.4	23	7				2			
39	4.4	3.6	4.2	10	6		1	1	2	0	1	
42	4	3.6	3.8	18	7	4	5	3	3	1	1	3
43	4.6	4.6	4.4	14	7	2	4	2	2	1	1	2
45	3.4	4.4	4.6	18	6	2	3	2	3	1	1	0
34	4.8	4.2	4	25	8	2	3	1	1	0	1	1
18	2.4	4	3.6	15	5	2	3	1	1	0	1	2
28	3.2	3.8	3.4	18	7	3	5	4	2	2	1	0
44	4	4.2	3.8	23	9	3	4	2	3	1	1	2
44	4.6	4.6	4.2	37	11	3		4	3	2	1	1
45	3.8	3.6	4.4	20	6	1	4	3	2	1	1	3
38	4	4.4	5	19	8	2	2	2	2	1	1	0
42	4	4.6	4.6	27	9	3	4	3	3	1	1	2
45	4.4	4.2	4.2	44	9				3	0	1	
33	4	3.8	4	17	4	1	4	4	4	1	1	1
48	3.6	4	3.2	22	8	1	3	2	2	1	1	2
38	4	3.8	4.2	19	6	2	4	2	2	1	1	3
46	4	4	4	20	6	1	5	2	3	1	1	2
28	4.2	5	4.6	28	7	4	4	3	2	1	1	0
36	3.6	3.8	3.4	14	5	3	4	3	2	1	1	2
45	3.8	4.6	4.6	27	5	2	4	3	2	1	1	2
46	3.8	4.8	4.4	25	6	3	5	5	3	0	1	2
41	4	4.4	4.6	41	8	3	5	4	3	1	1	1
28	4	4	3.2	28	5	3	3	3	2	0	3	1
34	3.6	3.6	3.6	26	5	3	4	3	2	2	2	1
34	3.6	3.6	4.2	21	7	3	4	3	2	1	3	3
32	3.4	3.8	5	21	8	3	5	3	2	0	1	2
39	3.8	4	4.6	37	6	4	5	3	3	2	1	1
30	3.8	3.6	3.4	13	4	2	5	2	2	2	1	2
36	3.6	4.4	3.8	8	5				2			
21	4	3.8	3	15	5	2	4	3	2	1	3	3
43	3.6	4.6	4.8	32	7	2	4	3	4	1	1	2
30	3.8	4	3.8	19	7	2	4	2	2	1	3	2
32	3.6	4.2	4	37	9	2	3	2	3	1	1	0
44	3.6	4	4.2	38	8	2	5	2	2	1	1	1
43	3.4	4.2	4.4	28	8	3	5	3	3	1	1	2
26	2.6	4	3.6	15	7	4	3	4	3	1	1	1
41	3.6	4	3.8	20	6	2	3	3	3	1	1	2

33	3.2	3.6	3.2	17	6	3	4	3	2	1	1	3
40	2.8	3.6	3.8	21	4	3	5	3	4	0	1	2
42	4.4	3.6	4	22	4	3	5	3	4	4	3	4
35	3.6	4.4	3.8	18	7	3	4	3	2	0	1	0
35	3.6	4	4	25	8	2	4	2	3	1	1	2
36	2.8	4.6	4	16	7	3	4	4	3	2	1	3
27	3.4	3.4	4	7	4	2	5	4	2	2	1	1
44	3.6	4.6	4.2	9	4	1	5	2	2	1	1	3
49	3.2	3.8	4.4	25	7	2	5	2	3	1	1	0

HEARD ALPHA	ALPHA IDENTITY 1	ALPHA IDENTITY 2
2	2	3
2	2	1
2	2	3
2	2	3
2	2	2
2	2	2
2	2	3
2	1	1
2	2	3
2	2	2
1	2	3
2	2	3
2	2	3
2	2	3
1	1	2
1	2	3
2	1	3
2	1	3
2	1	3
1	1	2
2	2	2
2	1	2
1	2	3
2	2	3
1	1	1
2	1	2
2	2	3
2	2	3
2	1	3
2	1	1
2	1	3
2	2	2
2	2	3
2	2	3
1	2	3
2	2	3
1	1	3
2	2	2
1	2	3
1	1	2
2	2	1
1	1	2
2	2	3
2	1	1
2	2	2
2	2	2

2	2	2
2	2	2
2	2	3
2	2	3
1	2	2
2	1	2
2	2	3
1	1	3
2	1	1
2	1	2
2	2	3
2	2	2
1	1	1
2	2	3
2	2	3
2	2	3
2	2	2
2	2	3
2	2	2
2	2	2
2	1	2
2	2	2
2	2	3
2	2	3
2	1	2
2	1	1
2	2	3
2	2	2
1	2	3
2	1	2
2	1	2
2	1	2
2	2	3
2	2	2
2	2	2
1	1	2
1	2	3
2	1	2
2	2	1
2	2	3
2	1	3
2	2	3
2	2	3
2	2	3
2	1	2
2	2	3
2	2	3
2	1	3
2	2	2

2	2	2
2	2	3
2	1	2
2	2	3
2	2	3
2	2	3
2	2	3
2	1	2
2	2	3
2	2	3
2	2	3
2	2	3
1	1	3
2	2	3
1	1	1
2	2	3
2	2	3
1	2	2
2	2	2
2	2	2
2	1	2
2	2	2
2	2	3
2	1	2
1	2	3
1	1	3
2	1	1
1	2	2
2	2	3
2	1	1
2	2	3
2	2	2
2	2	3
2	2	3
2	2	3
2	2	3
2	2	3
1	1	2
1	1	1
2	2	2
2	1	2
2	2	2
2	2	2
2	1	2
1	2	2
2	1	2
2	2	3
2	2	2
2	2	3

1	1	1
2	2	3
2	2	2
2	2	2
2	2	3
1	1	1
2	2	3
2	2	3
2	2	3
2	2	2
2	2	3
2	2	3
2	2	2
2	2	2
2	2	3
2	2	2
2	2	3
1	2	3
2	1	3
2	2	2
2	2	2
2	2	3
2	2	3
2	2	3
1	2	3
2	2	3
2	1	3
2	2	2
1	1	3
2	2	3
1	2	2
2	2	3
1	1	3
2	2	3
2	2	3
2	2	3
2	2	3
2	2	2
1	1	1
2	1	2
2	1	2
2	2	3
2	1	2
2	2	1
2	1	2
2	2	2
2	2	3
2	1	2
2	1	1

2	1	3
2	2	3
2	2	3
2	2	2
2	2	3
2	2	3
2	2	3
2	2	2
2	2	3
2	2	3
2	2	2
2	1	2
2	2	2
2	1	2
2	1	2
2	1	3
2	2	3
2	2	3
2	2	3
2	2	3
2	2	3
1	1	2
2	1	3
2	2	3
1	1	2
2	2	3
2	2	3
2	1	1
2	2	2
2	1	2
2	1	2
2	2	3
2	2	3
2	2	2
1	1	3
2	2	2
2	2	3
2	2	3
2	2	3
2	2	3
1	2	3
2	1	3
2	1	1
2	2	3
2	2	3
2	2	3
2	1	2
2	1	2
2	1	2

2	2	2
2	2	2
2	2	2
2	1	3
2	2	3
2	2	3
2	1	2
1	2	3
2	2	3
2	1	2
2	2	2
2	2	2
2	1	3
2	2	2
1	2	3
2	2	3
2	2	3
2	2	3
2	2	3
2	1	3
2	2	2
2	2	3
2	2	2
2	2	3
2	2	2
2	2	1
2	2	3
2	2	1
2	2	3
2	2	1
2	2	3
2	2	3
1	2	3
1	2	3
2	2	3
2	2	2
2	2	3
2	2	2
2	2	3
2	2	3
2	1	3
2	2	2
2	1	2
2	2	3
2	1	1
2	2	3
2	2	3
1	1	2
2	2	2

2	2	3
2	2	3
2	2	3
2	2	2
2	1	2
2	1	2
2	1	2
2	1	2
2	2	3
2	2	2
2	2	3
2	2	3
2	2	3
2	2	2
2	2	3
1	2	2
1	2	1
2	2	3
2	2	2
2	2	3
2	2	1
1	1	3
2	1	3
1	1	2
2	2	3
2	1	2
1	2	3
2	2	3
1	2	3
2	1	2
2	2	3
2	1	2
2	2	2
2	2	2
2	2	3
2	1	2
2	2	3
2	2	3
2	2	3
2	2	2
2	2	2
1	2	2
2	2	2
2	2	3
2	1	2
2	1	2
2	2	3
2	2	2
2	2	3

1	2	3
1	1	2
2	2	2
1	2	3
2	2	3
2	1	2
2	2	3
2	2	3
1	1	1
2	2	3
2	2	2
2	2	2
2	1	2
2	1	2
2	2	2
1	1	1
2	2	3
2	2	3
1	2	3
2	2	3
2	2	3
2	2	3
1	1	3
2	2	2
2	2	3
2	1	2
2	2	3
2	1	2
2	1	1
2	2	2
2	2	3
2	2	3
2	2	3
1	2	3
1	1	3
2	1	1
2	2	2
2	1	2
2	1	2
2	2	3
2	2	1
2	2	3
2	2	2
2	2	3
1	2	3
2	2	3
1	1	2
2	2	2
2	1	1

1	2	2
2	2	3
2	1	1
2	1	2
2	1	2
2	2	3
2	2	2
2	2	2
1	1	3
